# Bone Formation in 2D Culture of Primary Cells

**DOI:** 10.1002/jbm4.10701

**Published:** 2022-12-13

**Authors:** Edward L. Mertz, Elena Makareeva, Lynn S. Mirigian, Sergey Leikin

**Affiliations:** ^1^ Eunice Kennedy Shriver National Institute of Health and Human Development National Institutes of Health Bethesda MD USA

**Keywords:** CHONDROCYTES, COLLAGEN, MATRIX MINERALIZATION, MC3T3, OSTEOBLASTS, OSTEOCYTES

## Abstract

Relevance of mineralized nodules in two‐dimensional (2D) osteoblast/osteocyte cultures to bone biology, pathology, and engineering is a decades old question, but a comprehensive answer appears to be still wanting. Bone‐like cells, extracellular matrix (ECM), and mineral were all reported but so were non‐bone‐like ones. Many studies described seemingly bone‐like cell‐ECM structures based on similarity to few select bone features in vivo, yet no studies examined multiple bone features simultaneously and none systematically studied all types of structures coexisting in the same culture. Here, we report such comprehensive analysis of 2D cultures based on light and electron microscopies, Raman microspectroscopy, gene expression, and in situ messenger RNA (mRNA) hybridization. We demonstrate that 2D cultures of primary cells from mouse calvaria do form bona fide bone. Cells, ECM, and mineral within it exhibit morphology, structure, ultrastructure, composition, spatial–temporal gene expression pattern, and growth consistent with intramembranous ossification. However, this bone is just one of at least five different types of cell‐ECM structures coexisting in the same 2D culture, which vary widely in their resemblance to bone and ability to mineralize. We show that the other two mineralizing structures may represent abnormal (disrupted) bone and cartilage‐like structure with chondrocyte‐to‐osteoblast transdifferentiation. The two nonmineralizing cell‐ECM structures may mimic periosteal cambium and pathological, nonmineralizing osteoid. Importantly, the most commonly used culture conditions (10mM β‐glycerophosphate) induce artificial mineralization of all cell‐ECM structures, which then become barely distinguishable. We therefore discuss conditions and approaches promoting formation of bona fide bone and simple means for distinguishing it from the other cell‐ECM structures. Our findings may improve osteoblast differentiation and function analyses based on 2D cultures and extend applications of these cultures to general bone biology and tissue engineering research. Published 2022. This article is a U.S. Government work and is in the public domain in the USA. *JBMR Plus* published by Wiley Periodicals LLC on behalf of American Society for Bone and Mineral Research.

## Graphical abstract

Primary mouse calvarial cells within the same 2D culture form multiple materials: genuine bone, disrupted bone, nonmineralizing osteoid‐like, unmineralized cambium‐like, and partly mineralized cartilage‐like cell‐ECM structures. Unless these structures are distinguished, culture mineralization may be a misleading representation of osteoblast differentiation, particularly in the presence of β‐glycerophosphate.

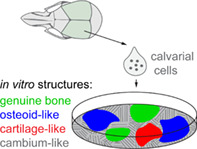

## Introduction

Debated for many decades, the physiological relevance of in vitro cell culture models for bone biology and pathology studies is still an enigma. Bone is a complex organ with structural, mechanical, endocrine, hematopoietic, and other functions whose regulation involves interactions between multiple organs.^(^ ^1^, ^2^, ^3^ ^)^ Nonetheless, studies of bone biology and pathology at cellular and molecular level often require explant or cell cultures that do not have these functions and mimic only a subset of bone properties.^(^ ^4^, ^5^, ^6^ ^)^ Although simplification is a feature of any model, the problem here is the lack of consensus on which bone properties need to be mimicked for a broader relevance of bone cell cultures.

Cell culture applications range from bone tissue engineering to mechanistic studies of bone cell functions.^(^ ^4^, ^5^, ^6^, ^7^, ^8^, ^9^, ^10^, ^11^ ^)^ One of the simplest and most common applications is mineralization assays for osteoblast differentiation.^(^ ^4^, ^12^, ^13^, ^14^, ^15^, ^16^ ^)^ For instance, effects of mutations, gene knockout/knockdown, and different compounds on osteoblasts are assessed from overall mineral deposition or formation of mineralized nodules in cell culture^(^ ^8^, ^14^, ^15^ ^)^ revealed by staining the mineral with Alizarin red^(^ ^17^, ^18^ ^)^ or Von Kossa.^(^ ^19^ ^)^ With some variations, this assay is used for primary cells from murine and human bones, bone marrow stromal cells (BMSCs), induced pluripotent stem cell (iPSC)‐derived cells, and immortalized osteoblast cell lines.

Although hundreds of papers published each year study osteoblasts and osteocytes in culture, interpretation of the results in terms of physiologically relevant differentiation and function of these cells is debatable. On one hand, a few features consistent with in vivo bone have been reported for some cultures, including mineral crystals,^(^ ^20^, ^21^ ^)^ collagen fiber and cell morphology,^(^ ^20^, ^22^, ^23^, ^24^ ^)^ and gene expression.^(^ ^8^, ^24^ ^)^ On the other hand, non‐bone‐like matrix, poor survival of matrix‐embedded cells, artificial mineralization induced by cell necrosis and β‐glycerophosphate (βGP), and other significant differences from in vivo bone have also been observed.^(^ ^25^, ^26^, ^27^, ^28^, ^29^ ^)^ A part of the problem is the lack of detailed and systematic investigations of different types of structures formed in culture, which would integrate structural, ultrastructural, and functional characterization of cells, extracellular matrix (ECM), and mineral. Not surprisingly, disparate published observations that used different cells and media as well as different criteria for bone‐like structures and mineralization produced inconsistent conclusions.

To address the problem of physiological relevance, we reexamined various mineralized and unmineralized cell‐ECM structures formed by cultured primary osteoblasts from murine calvaria and murine calvarial osteoblast cell line MC3T3‐E1. Specifically, we investigated how these in vitro structures compare with in vivo bone and surrounding tissue by integrating multiple techniques at different structural and functional levels. From this comparison, we identified which of these structures result from normal, physiological differentiation and function of osteoblasts.

Here, we demonstrate that cultured primary osteoblasts form a mineralized cell‐ECM structure consistent with bona fide bone at all tested levels. Yet they also form multiple distinct types of other, previously uncharacterized structures that are more prevalent at common culturing conditions. The bona fide bone structure has lamellar ECM with in‐vivo‐like composition, structure, ultrastructure, and collagen and mineral densities. It is typically covered by osteoid and a cell‐ECM structure resembling cambium layer of calvarial periosteum. Its formation and growth in culture resembles in vivo intramembranous ossification. Its surface osteoblasts and embedded osteocytes are similar to calvarial cells in morphology and gene expression. These cells survive for weeks, enabling their long‐term studies. The other, more prevalent types of cell‐ECM structures formed in culture are highly variable and have fewer or no characteristic features of normal in vivo bone. They do not represent normal, physiological osteoblast differentiation and function, although they might be interesting and useful for studies of pathological conditions. Importantly, we also show that crucial differences between the physiological and other cell‐ECM structures are masked by artificial mineralization at 10mM βGP commonly used in osteogenic media. We believe that these findings and our simple recipes for growing and identifying physiologically relevant cell‐ECM structures in osteoblast cultures may have important implications for bone biology and bone engineering studies.

## Materials and Methods

### Animals

C57BL/6J (stock #000664), OC‐cre (B6.FVB‐Tg(BGLAP‐cre)1Clem/J, stock #019509), Ai14 (B6.Cg‐Gt(ROSA)26Sor^tm14(CAG‐tdTomato)Hze^/J, stock #007914), and Osx1‐GFP::Cre (B6.Cg‐Tg(Sp7‐tTA, tetO‐EGFP/cre)1Amc/J, stock #006361) mice were purchased from The Jackson Laboratory (Bar Harbor, ME, USA) and maintained on the C57BL/6J background. For most experiments, primary cells were extracted from parietal bones of 3–7‐day‐old C57BL/6J pups. To visualize cells expressing osterix, primary cultures were established from hemizygous Osx1‐GFP::Cre pups. To visualize osteoblasts and cells derived from them, primary cultures were established from parietal bones of 3–7‐day‐old double‐hemizygous progeny of Ai14 × OC‐cre crosses, in which expression of Cre‐recombinase driven by osteocalcin promoter activates tdTomato transgene. Animal care and experiments were performed in accordance with a protocol approved by the Eunice Kennedy Shriver National Institute of Child Health and Human Development Animal Care and Use Committee.

### Cell cultures

Primary cells were extracted from parietal bones, seeded, and cultured without passaging as described.^(^ ^30^ ^)^ To prevent excess accumulation of unfolded/misfolded procollagen in the endoplasmic reticulum (ER)^(^ ^31^, ^32^, ^33^, ^34^ ^)^ disrupting osteoblast differentiation, the culture medium was supplemented with 100μM ascorbic acid 2‐phosphate (Asc2P; Sigma‐Aldrich, St. Louis, MO, USA) at all times. The cells were either (i) seeded at 10,000 cells/cm^2^ and cultured in a standard CO_2_ incubator with atmospheric oxygen (~20% O_2_, 5% CO_2_, ~75% N_2_) or (ii) seeded at 2000 cells/cm^2^, expanded to confluency (~5–7 days) in a tri‐gas incubator with reduced oxygen (5% O_2_, 5% CO_2_, ~90% N_2_) to stimulate proliferation,^(^ ^35^, ^36^ ^)^ and subsequently cultured in the standard incubator to stimulate mineralization. Cells were cultured in plastic 6‐well, 12‐well, or 24‐well plates at 37°C in a growth medium containing α‐minimal essential medium (αMEM)+Glutamax (32571; Thermo Fisher Scientific, Waltham, MA, USA), Asc2P, 1% Pen‐Strep (Corning, Corning, NY, USA), and 10% fetal bovine serum, pretested for supporting osteoblastic differentiation (Valley Biomedical, Winchester, VA, USA; Cat#BS3033, Lot#2C0550; GeminiBio, West Sacramento, CA, USA; GemCell™, Lot #A83F821). Media were replaced every 48–72 hours.

Unlike primary cells, MC3T3‐E1 murine calvarial cells (subclone 4; American Type Culture Collection [ATCC], Manassas, VA, USA; ATCC CRL‐2593) were expanded and maintained without supplemental Asc2P or ascorbic acid, either in ascorbic‐acid‐free αMEM (A10490, Thermo Fisher Scientific) as recommended by ATCC or in regular αMEM (32571; Thermo Fisher Scientific). In both media, the absence of active ascorbic acid enabled the cells to remain in a similar undifferentiated state (Fig. S1). Like others,^(^ ^37^, ^38^, ^39^ ^)^ we found that ascorbic acid contained in αMEM‐32571 was inactivated and had to be supplemented with fresh 100μM Asc2P for initiating osteoblastic differentiation.^(^ ^33^ ^)^ MC3T3‐E1 cells were seeded at ~40,000 cells/cm^2^ and cultured in the standard CO_2_ incubator with atmospheric oxygen.

Treatments of both primary and MC3T3 cells with rapamycin (Rap) or βGP were started at confluency in the standard CO_2_ incubator. Ten nanomolar (10nM) Rap was added to freshly prepared media during the first week after confluency (beyond which we observed no qualitative Rap effects on the appearance and content of cell‐ECM structures). One millimolar (1mM) or 10mM βGP was added to the media starting at confluency for the entire duration of the experiments. To examine *Fgf23* expression, primary cells were treated with 30nM 1α,25‐dihydroxyvitamin D3 (Sigma‐Aldrich: D1530) for 2 days starting at 12 days post‐confluency (once partial mineralization was observed). Media supplemented with vitamin D3 were refreshed on the second day of treatment.

### Sectioning

To cross‐section different cell‐ECM structures, 2–3‐mm pieces were dissected with a scalpel from layers grown in culture or bones under a microscope. All samples were kept hydrated and imaged in top view transmission with 0.63×, 4×, and 10× objectives to identify the precise location of desired cell‐ECM structures before demineralization. Femoral bone samples were cut out from posterior mid‐diaphysis cortex with a thin diamond disk. All samples were fixed, demineralized (as needed), and embedded in a well‐defined orientation relative to the embedding block in an aqueous solution of 20% 360 kDa and 10% 10 kDa polyvinylpyrrolidone (PVP; Sigma‐Aldrich), or Super Cryoembedding Medium (SCEM) (Section‐Lab, Hiroshima, Japan), or Spurr's epoxy resin. Sample cross‐section images were mapped onto top view images to select desired structures. A Vibrotome Deluxe cryotome (Hacker Instruments, Winnsboro, SC, USA) with a tungsten carbide D‐profile knife and a Leica EM UC7 ultramicrotome with a diamond knife (Leica Microsystems, Inc., Buffalo Grove, IL, USA) were used.

For light microscopy, Raman microspectroscopy, and in situ messenger RNA (mRNA) hybridization, samples were fixed for 16–24 hours at room temperature in 1% or 2% freshly prepared solution of formaldehyde in phosphate‐buffered saline (PBS) (made from 16% methanol‐free formaldehyde solution sealed in ampules; Pierce, Rockford, IL, USA) or PBS + 0.5mM CaCl_2_ (PBS‐Ca) to preserve mineral. For electron microscopy, samples were fixed for 0.5 or 12 hours at room temperature with fresh 2.5% glutaraldehyde in 0.13M Na‐cacodylate (pH 7.4) or 0.13M Na‐cacodylate +2mM Na‐phosphate +0.5mM CaCl_2_ (to preserve mineral). As needed, fixed samples were demineralized in 0.1M ethylenediamine tetraacetic acid (EDTA). To remove molecules masking collagen fibers in electron microscopy, some samples were demineralized before glutaraldehyde fixation.

Cryosections of PVP‐embedded samples (5–10 μm) were mounted onto glass or quartz slides in PBS‐Ca or in Prolong Diamond aqueous mounting medium (Invitrogen, Carlsbad, CA, USA) with or without 4′, 6‐diamidino‐2‐phenylindole (DAPI). Prior to mounting onto the slides, some cryosections of SCEM‐embedded samples (5 μm) were attached to adhesive cryo‐film (Section‐Lab) and postfixed for 30–60 minutes at room temperature with freshly prepared 2% formaldehyde in PBS‐Ca followed by several minutes in 70% ethanol. The latter procedure helped to prevent loss of bone sections during in situ mRNA hybridization.

### Light microscopy

Low magnification (0.63×, 4×, 10×, and 20× objectives) top view and cross‐section imaging was performed on AE2000 (Motic, Schertz, TX, USA), Senterra (built on Olympus BX51 frame; Bruker Optics Inc., Billerica, MA, USA), EVOS FL AUTO (Life Technologies, Inc., Grand Island, NY, USA), Olympus MVX10 (Olympus America, Center Valley, PA), and Axio Observer.Z1/7 (Carl Zeiss Microscopy, Inc., Dublin, CA, USA) microscopes in transmission, phase‐contrast, dark‐field polarized, and fluorescence modes. For some time‐lapse imaging, cells were seeded on micro‐dishes with grid marks (Ibidi GmbH, Gräfelfing, Germany) and imaged at the same grid areas. Higher resolution, confocal imaging was performed with a 63×/1.4 NA objective on a LSM780 microscope (Carl Zeiss).

Importantly, different types of cell‐ECM structures could be identified in top‐view bright‐field images with a 10×/0.3 NA objective in either basic cell culture or more advanced microscopes. Objectives of 4× and 20× were beneficial for larger areas and finer details, respectively, but provided inferior clarity for distinguishing different types of structures. Reliable structure identification required a focused and centered condenser with the numerical aperture set slightly below that of the objective, phase ring removal from the condenser, and sample mounting in physiological salt solution (mounting media with higher refractive index provided inferior clarity). The mineralized regions appeared darker and had more prominent three‐dimensional (3D) texture than adjacent nonmineralized regions. After white color balance correction, thicker ECM layers with high protein density acquired more pronounced brownish hue (caused by blue light absorption), so that they could be distinguished from thinner and/or lower density ECM layers.

### Staining

To visualize mineral apposition, cultures were stained for 2 hours with 16 μg/mL calcein (Sigma‐Aldrich) in culture media at 20 days post‐confluency, washed, cultured for 7 more days, stained with 50 μg/mL Alizarin complexone (Sigma‐Aldrich) for 2 hours, washed, and cultured for 2 more weeks. The cultures were then fixed as described in Sectioning and imaged. To visualize alkaline phosphatase activity, cultures were fixed and stained with SIGMAFAST™ BCIP®/NBT (Sigma‐Aldrich) according to the manufacturer's protocol. To visualize osteocyte processes and cellular cytoskeleton in cultures and bone, actin filaments were stained with 200nM Sir‐Actin probe (Cytoskeleton, Inc., Denver, CO, USA) according to the manufacturer's protocol. Actin staining in live cells was enhanced with 1–10μM Verapamil (Cytoskeleton). Cell nuclei were stained with DAPI (1–10 μg/mL; Thermo Fisher Scientific; fixed samples) or Hoechst 33342 (0.1–1 μg/mL; Thermo Fisher Scientific; live cultures). Some fixed cryosections were demineralized in 0.1M EDTA and stained with saturated toluidine blue solution in 0.2× PBS for 5 minutes. Expression of mRNA was visualized in 5‐μm cryosections with RNAScope Fluorescent Multiplex V2 assay (Advanced Cell Diagnostics, Newark, CA, USA) according to the manufacturer's protocol.

### Image processing and analysis

Bright‐field transmission images were corrected for white color balance and intensity variations by linear rescaling of intensities of 8‐bit red, green, and blue channels to the value of 220 in blank hydrating solution near each sample. Fluorescence images of stains other than DAPI were overlaid onto bright field images by linear superposition of red, green, and blue channels. To better visualize DAPI‐stained cell nuclei in overlaid bright‐field and fluorescence images, blue and green DAPI channels were nonlinearly enhanced by gamma correction with γ = 2, and the bright‐field intensities were reduced proportionally to the DAPI intensity at each pixel. Image processing and analysis were performed with ImageJ software (NIH, Bethesda, MD, USA; https://imagej.nih.gov/ij/). Final images were trimmed, labeled, and assembled for publication with Adobe Illustrator and Photoshop (Adobe, San Jose, CA, USA).

### Electron microscopy

For transmission electron microscopy (TEM), glutaraldehyde‐fixed culture and bone samples were postfixed in 2% OsO_4_, processed into Spurr's epoxy, sectioned to 90 nm, stained with uranyl acetate and lead citrate, and examined in a JEOL 1400 electron microscope (JEOL USA, Inc., Peabody, MA, USA). Volume fractions of collagen fibers were analyzed within representative 0.5–3‐μm regions away from cellular lacunas and processes. In well‐ordered ECM, the volume fractions were evaluated from the area occupied by fibers within regions with fibers perpendicular to the section plane. When such regions could not be selected in less ordered cell‐ECM structures, the volume fraction was corrected for the tilt of each fiber as described in Fig. S2*A*. The fused fibers fraction was evaluated as the ratio of volume of fused fibers to the volume of all fibers within section regions where fused fibers could be clearly distinguished.

### Raman microspectroscopy

Backscattered Raman spectra were collected on a Senterra confocal Raman microscope (Bruker Optics) at 532 nm excitation wavelength (20 mW laser) through a 40×/0.95 NA objective (Olympus America), a 50μm pinhole and a 400 grooves/mm grating. Briefly, 8‐μm or 10‐μm cryosections were cut from PVP‐embedded cell‐ECM structures formed in culture (perpendicular to the culture surface), parietal bones of 6‐week‐old mice (perpendicular to the bone surface), and posterior mid‐diaphysis femoral cortex from 16‐week‐old mice (perpendicular to the periosteal surface and parallel to the femur axis). The sections were rinsed to remove the embedding medium, hydrated in PBS‐Ca and mounted on 1‐mm fused quartz slides with 0.16‐mm fused quartz coverslips (Esco Optics, Oak Ridge, NJ, USA). Points of interest (POIs) for spectra acquisition were selected in bright‐field, dark‐field polarized, and sometimes fluorescence modes of the microscope. Each POI was photobleached for 15–60 seconds (to reduce autofluorescence) followed by averaging of two spectra with circularly polarized laser and no emission polarizer or 10 spectra with linearly polarized laser and a similarly oriented emission polarizer. The spectra were normalized for throughputs of the excitation and emission channels and corrected for the dark signal, cosmic ray spikes on the detector, bulk water, quartz, and background (polynomial line). The sample composition at each POI was quantified from baseline‐corrected integral intensities of the following peaks in circularly polarized spectra: CH‐stretching at 2830 cm^‐1^ to 3028 cm^‐1^ (total organic content, referred to as organics), amide III at 1212 cm^‐1^ to 1306 cm^‐1^ (collagen), ν_1_ of hydroxyapatite phosphate at ~959 cm^‐1^ (mineral), and hydroxyapatite carbonate at ~1071 cm^‐1^ (carbonate). The POIs in mouse bones were selected within fully mineralized lamellar bone regions formed more than 3 weeks before animal euthanasia, which were identified based on polarized and fluorescence images of the sections from calcein‐injected mice as described.^(^ ^40^ ^)^

### qPCR

RNA was purified with a Direct‐zol kit (Zymo Research, Irvine, CA, USA) and reverse transcribed with a random hexamer primer mix using SuperScript III kit (Thermo Fisher Scientific). Relative mRNA transcription was measured on a 7500 Fast Real Time PCR system (Applied Biosystems, Foster City, CA, USA) with TaqMan gene expression assays (Applied Biosystems) for *Acan* (Mm00545794_m1), *Alpl* (Mm00475834_m1), *B2m* (Mm00437762_m1), *Bglap* (Mm03413826_mH), *Bril* (Mm00804741_g1), *Col1a1* (Mm00801666_g1), *Col2a1* (Mm01309565_m1), *Col10a1* (Mm00487041_m1), *Dmp1* (Mm01208363_m1), *Fgf23* (Mm00445621_m1), *Gapdh* (Mm99999915_g1), *Hprt1* (Mm01545399_m1), *Ibsp* (Mm00492555_m1), *Mepe* (Mm02525159_s1), *Phex* (Mm01166563_m1), *Runx2* (Mm00501584_m1), *Sost* (Mm00470479_m1), *Sox9* (Mm00448840_m1), *Sp7* (Mm00504574_m1), and *Spp1* (Mm00436767_m1). *Gapdh*, *Hprt1*, and *B2m* were used as endogenous controls (EC) for calculating the values of ΔΔC_T_.

$${\text{ΔΔC}}_{\text{T}}={\left({\text{C}}_{\text{T}}\;-{\text{C}}_{\text{T}}\left(\text{EC}\right)\right)}_{\text{S}}\;-{\left({\text{C}}_{\text{T}}-{\text{C}}_{\text{T}}\left(\text{EC}\right)\right)}_{\text{S0}}$$

Here C_T_ is the qPCR signal threshold, C_T_(EC) = [C_T_(*Gapdh*) + C_T_(*Hprt1*) + C_T_(*B2m*)]/3, S = sample, S0 = reference sample.

### Statistical analysis

Standard deviations (SDs) and probabilities of false positive findings (*p* values) were calculated based on at least three replicates. The corresponding number of replicates (*N*‐value) is indicated in each figure. The *p* values were calculated from a two‐tailed Student's *t* test following Shapiro‐Wilk test for normality and Brown‐Forsythe test for equal variance. The dispersion (spread) of collagen fiber diameter in TEM images was characterized by the square root of the distribution variance. The dispersion error was calculated from the standard error of the unbiased variance estimator.^(^ ^41^ ^)^

## Results

### General cell culture features

To examine bone‐related structures forming in vitro, we cultured parietal bone cells (PBCs) from C57BL/6J mouse calvaria and murine calvaria clone MC3T3‐E1 (subclone 4) in growth media with Asc2P (100μM) ± βGP (1mM or 10mM) or Rap (10nM). Because βGP is the most frequently used osteogenic media component^(^ ^15^, ^16^ ^)^ also known to cause artificial (non‐bone‐like) mineralization,^(^ ^4^, ^26^, ^42^ ^)^ we examined both common (10mM) and lower (1mM) βGP concentrations. We tested Rap effects on mineralization because we previously observed more consistent mineralization of primary PBC cultures in an unrelated study of a G610C mouse model of osteogenesis imperfecta.^(^ ^30^ ^)^

**Fig. 1:**
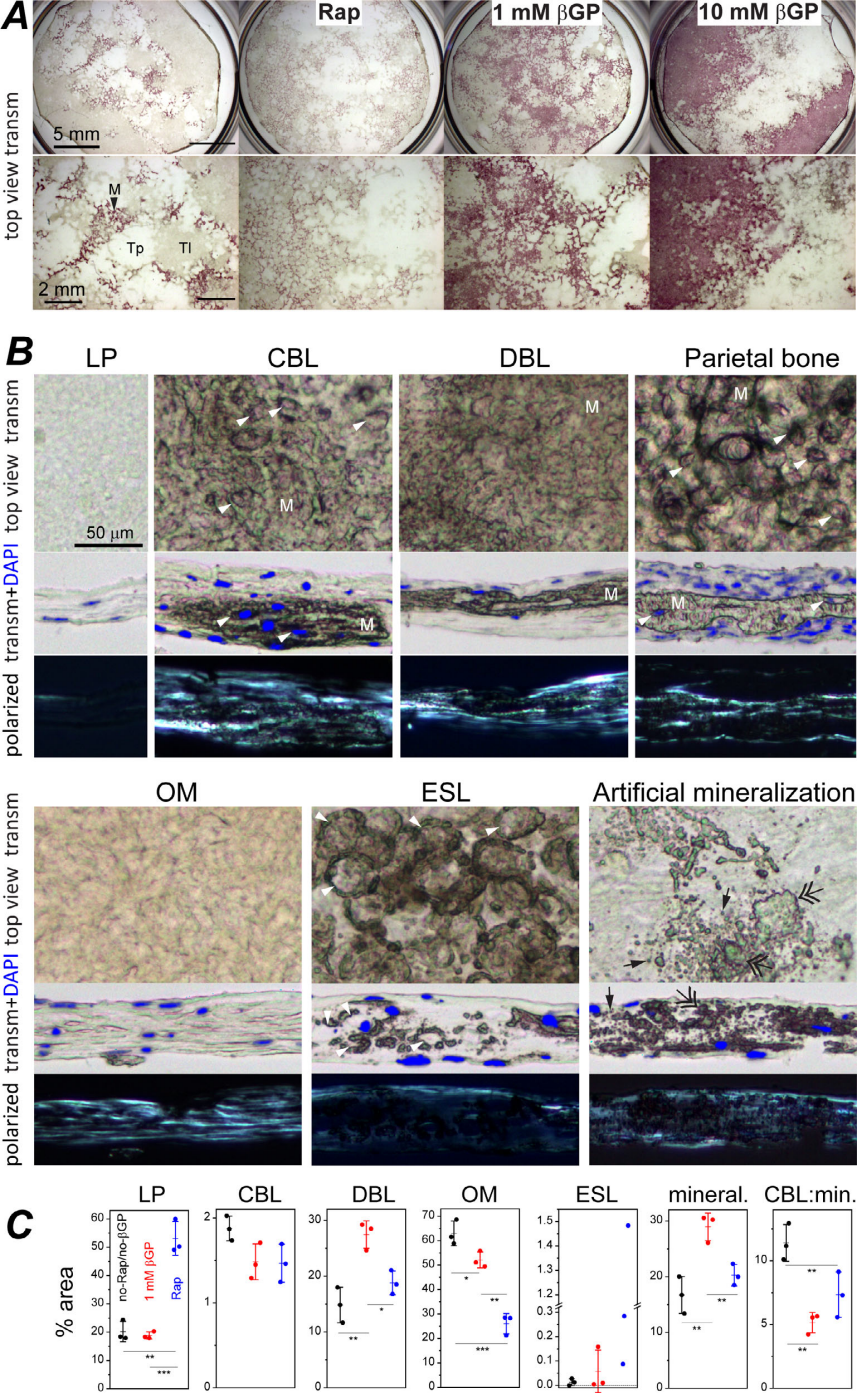
Cell‐ECM structures in cultures of primary parietal bone cells and in parietal bone. (*A*) Top view transmission images of cultures grown in media containing indicated supplements and stained with alizarin complexone (0.63× objective). Asc2P was present in all cultures. Zoomed‐in images (bottom) show transparent (Tp), translucent (Tl), and opaque, stained mineralized (M) patches. (*B*) Top view and cross‐section images of LP, CBL, DBL, OM, ESL, and artificially mineralized LP cell‐ECM structures in culture and of a parietal bone from a 5‐day‐old mouse with periosteal surface facing up (10×/0.3 NA objective). Mineralized matrix (M) is marked in CBL, DBL, and parietal bone. Arrowheads mark cell lacunas inside mineralized matrix in CBL and parietal bone and ovoid lacunas with perilacunar mineralization in ESL. Single and double arrows mark individual and merged mineral granules, respectively, which were formed by artificial mineralization. The cultures were grown without βGP or Rap (LP, OM, ESL), with Rap (CBL, DBL), or with 10mM βGP (artificial mineralization). Rap or 1mM βGP did not qualitatively alter the appearance of these cell‐ECM structures. Transmission cross‐section images (middle) are overlain with DAPI fluorescence of cell nuclei. Dark‐field, polarized images of the same areas (bottom) reveal well‐oriented collagen fibers (bright). Alternating darker and brighter stripes in CBL and OM indicate lamellar ECM. All polarized images were captured at the same microscope settings. All bright‐field transmission images were corrected for white balance and intensity variations without contrast enhancement (see Materials and Methods). ECM with high thickness and high protein density is distinguished by its brownish hue. Unstained mineralized ECM appears darker and has a more prominent 3D texture. (*C*) Area fraction occupied by individual structures and by all mineralized structures (CBL, DBL, and ESL) in a culture grown for 34 days with indicated supplements. Each data point represents a different culture well in the same experiment. Error bars represent standard deviations; *p < 0.05, **p < 0.01, and ***p < 0.001. βGP = β‐glycerophosphate; CBL = continuous bone‐like; DBL = discontinuous bone‐like; ESL = eggshell‐like; LP = loosely packed; M = mineralized; OM = osteoid‐mimicking; Rap = 10nM rapamycin; Tl = translucent unmineralized; Tp = transparent unmineralized.

**Fig. 2:**
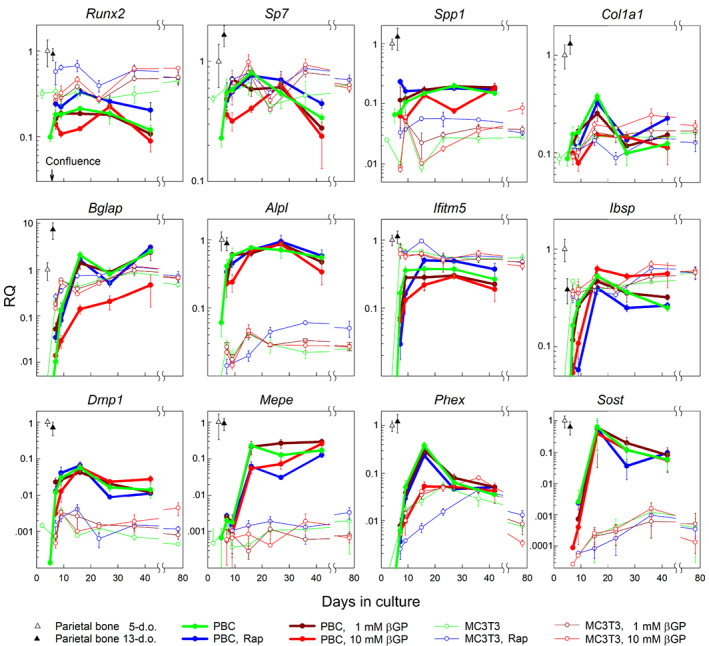
Transcription of key osteoblast genes in primary PBC cultures, MC3T3‐E1 cell cultures, and in vivo parietal bone from 5‐day‐old and 13‐day‐old mice. Cultures were grown in media containing indicated supplements. RQ of mRNA measured by qPCR represents 2^−ΔΔC^_T_ normalized to parietal bone from 5‐day‐old mice (using *B2m*, *Gapdh*, and *Hprt1* as housekeeping genes). Error bars are the standard deviations for *N* = 3 (culture) wells of PBCs pooled from multiple animals and *N* = 5 (bone) different animals. Reproducibility of the data in different cell preparations of primary PBCs and in different stocks of MC3T3‐E1 is demonstrated by Fig. S3. PBC = parietal bone cell; RQ = relative quantity.

Primary PBCs deposited mineral (Fig. 1*A*) concomitant with increasing expression of mature osteoblast (*Ibsp*, *Ifitm5*, and *Bglap*) and late osteoblast/osteocyte (*Dmp1*, *Mepe*, *Phex*, and *Sost*) marker genes (Fig. 2) within 2–3 weeks after reaching confluency. This was consistent with mineralization being driven by osteoblast maturation like it occurs in vivo.^(^ ^43^ ^)^ Although βGP and Rap were not required for mineralization of PBC cultures, they accelerated nucleation of mineralization, and βGP increased the mineralized area (Fig. 1*A*,*C*).

MC3T3‐E1 cells consistently deposited mineral within 5 weeks of culture only at 1mM βGP (Fig. S4*B*). Compared to PBCs, MC3T3‐E1 cells exhibited faster upregulation of *Ibsp*, *Ifitm5*, and *Bglap* and had lower expression of both *Alpl* essential for mineral formation^(^ ^44^, ^45^ ^)^ and *Dmp1*, *Mepe*, and *Sost* markers of late osteoblasts and osteocytes^(^ ^46^, ^47^ ^)^ (Figs. 2 and S3). The lower expression of *Alpl*, *Dmp1*, *Mepe*, and *Sost*, few osteocyte‐like cells embedded into mineralized cell‐ECM nodules, and deficient formation of the canalicular network by the embedded cells (Fig. S4*B*,*C*) pointed to MC3T3‐E1 cells being a limited model for bone formation in culture. Therefore, we performed only cursory analyses of MC3T3‐E1 cells described in Fig. S4 and instead focused on mineralized structures formed by primary cells.

**Fig. 3:**
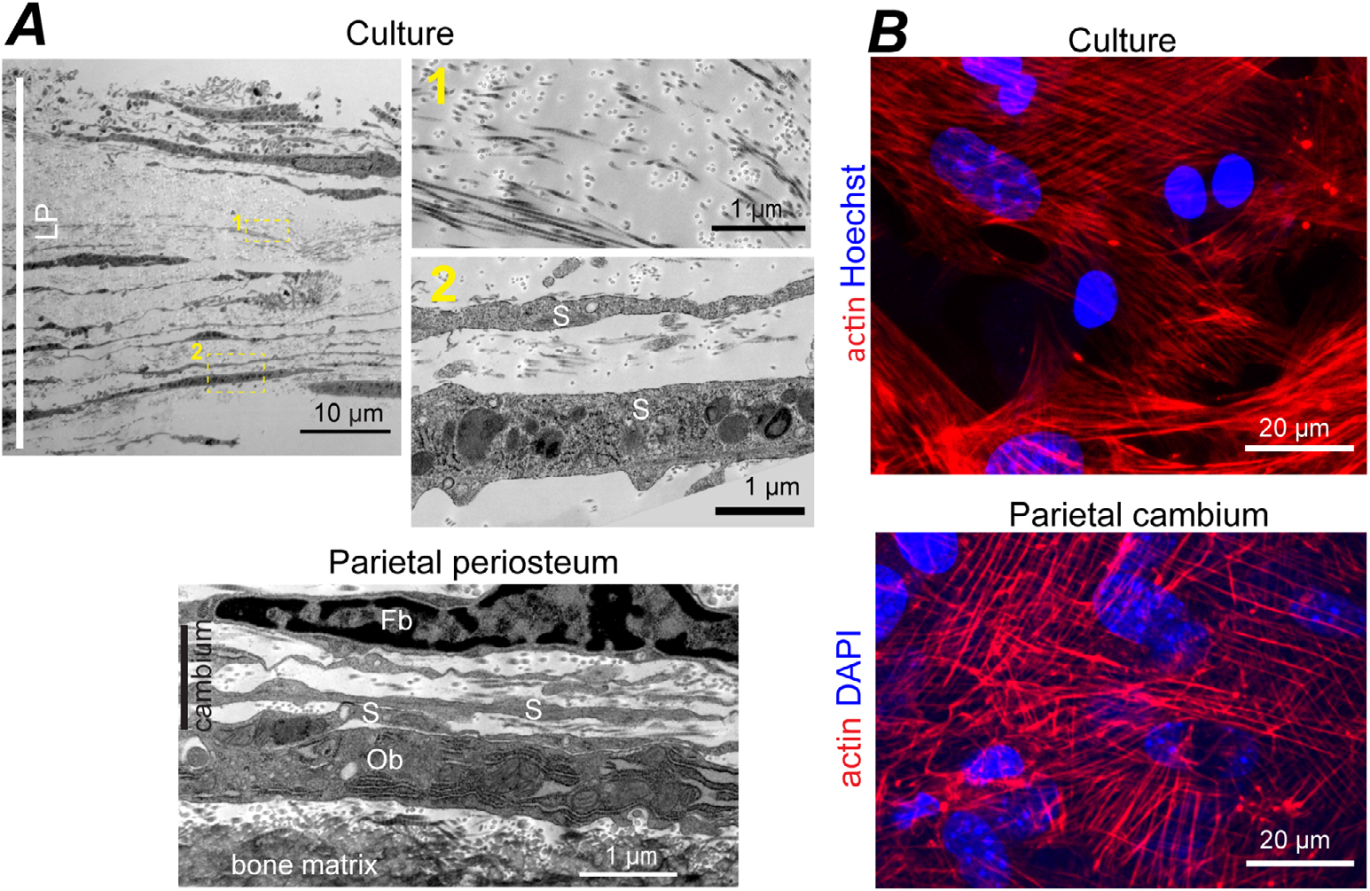
LP cell‐ECM structure and cambium layer of parietal bone periosteum. (*A*) Cross‐section TEM images of full‐thickness LP in primary PBC culture (top, no βGP or Rap) and parietal bone periosteum from a 5‐day‐old mouse (bottom). Boxes mark zoomed‐in regions shown in the correspondingly numbered panels. Fb, Ob, and S mark fibroblast‐like, osteoblast‐like, and spread‐out cells, respectively. (*B*) Confocal fluorescence images of actin stress fibers and cell nuclei in LP layer (top, no βGP or Rap) and cambium layer of parietal bone (bottom) from a 4‐day‐old mouse. The images are optical slices through the layers parallel to layer plane. LP = loosely packed.

**Fig. 4:**
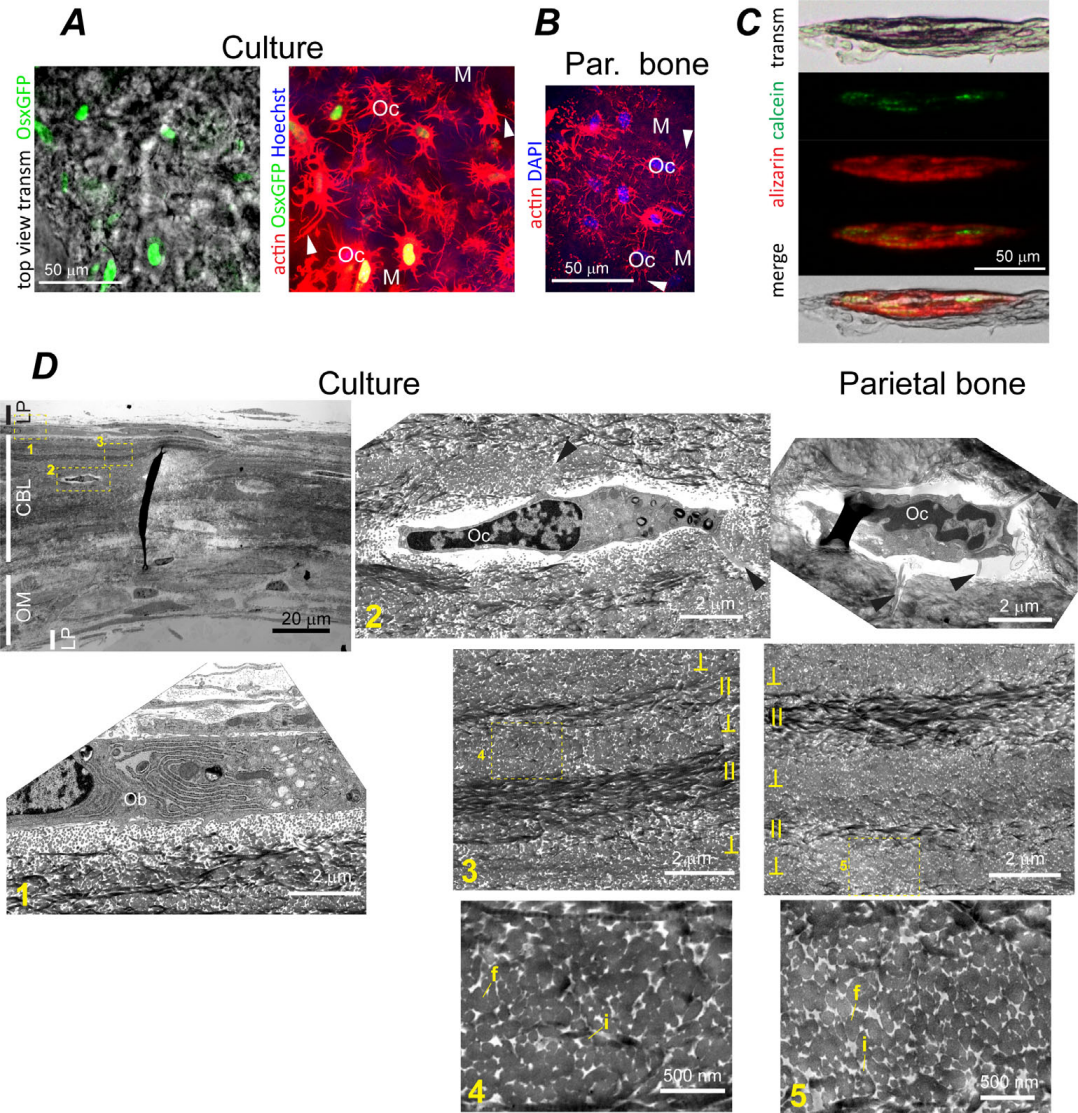
CBL structure in PBC cultures and parietal bone. (*A*) Top view transmission and confocal fluorescence images of CBL in live PBC culture from transgenic mice expressing a Cre::GFP construct in cells actively transcribing *Sp7*/osterix (no βGP or Rap). The left panel shows GFP signal (green) from cell nuclei overlaid with the transmission image. The right panel image of the same area (optically sliced through the middle of the mineralized CBL layer) shows the same GFP signal overlaid with actin cytoskeleton (red) and cell nuclei (blue). (*B*) Confocal fluorescence image showing actin cytoskeleton (red) and nuclei (blue) of osteocytes optically sliced through the middle of the mineralized layer of parietal bone from a 4‐day‐old mouse. In *A* and *B*, arrowheads point to processes of Ocs embedded into mineralized ECM (M, blue autofluorescence). (*C*) Transmission and wide‐field fluorescence images of a CBL cross‐section labeled by a calcein pulse at 3 weeks and by an alizarin complexone pulse at 4 weeks after induction of osteogenic differentiation (10nM Rap). (*D*) TEM images of primary PBC culture (no βGP or Rap) and parietal bone cross‐sections. Low resolution image (top left) shows layering of LP, CBL, and OM structures. Boxes mark zoomed‐in regions shown in the correspondingly numbered panels. Ob and Oc mark cells with osteoblast and osteocyte morphology, respectively. Arrowheads point to cell processes. Fused and individual collagen fibers are marked by letters f and i. Lamellas with collagen orientation approximately parallel and perpendicular to the section plane are marked by II and ⊥. CBL = continuous bone‐like; Oc = osteocyte‐like cell.

**Fig. 5:**
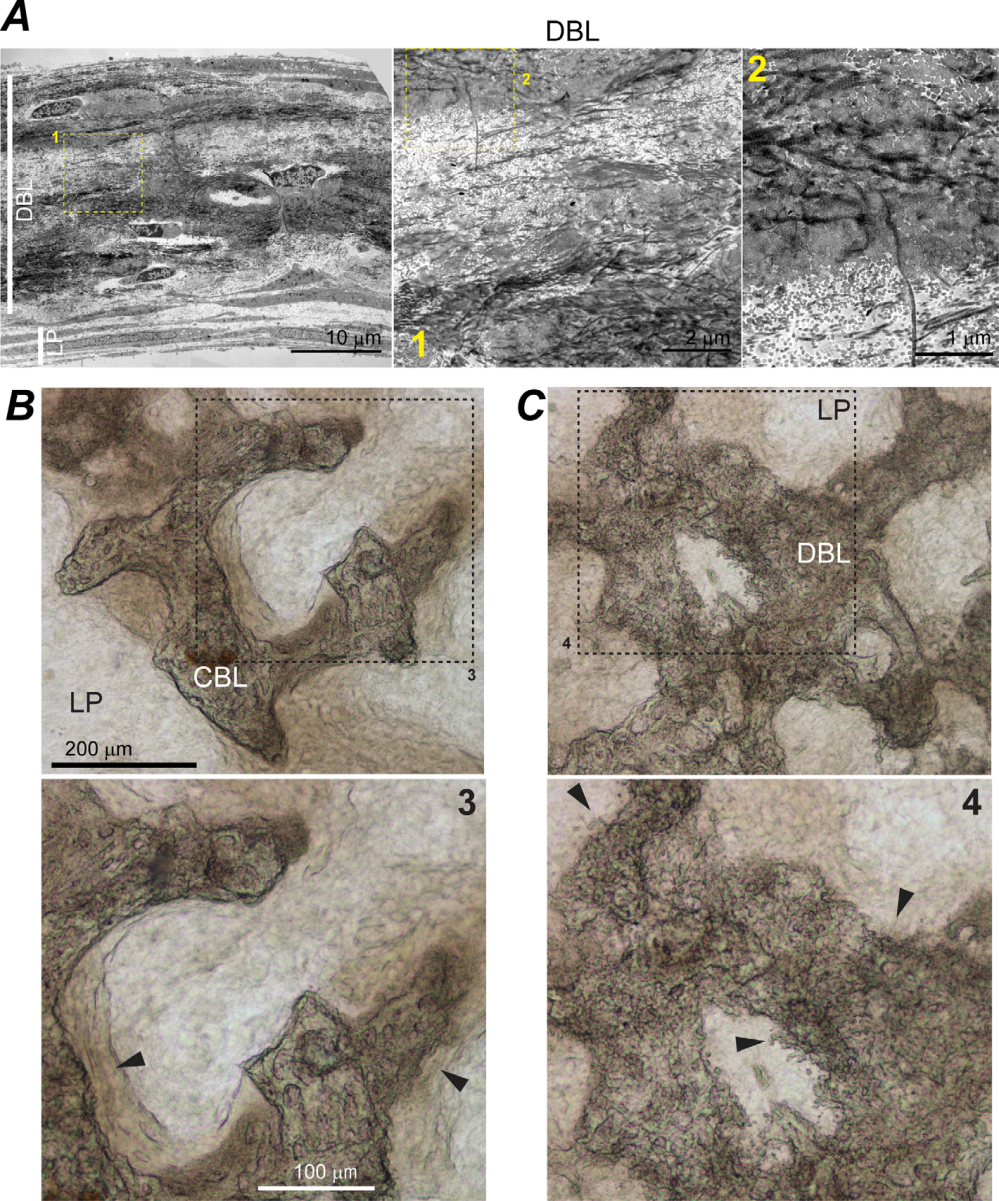
DBL and CBL structures in PBC cultures. (*A*) Low resolution TEM image (left panel) shows layering of DBL and LP structures in a cross‐section of a primary PBC culture (no βGP or Rap). The middle and right panels show zoomed‐in DBL regions marked with the correspondingly numbered boxes on the left panel. ECM mineralization occurred only within regions with smeared ultrastructure. The smearing was caused by uranyl acetate staining of negatively charged non‐collagenous proteins, which were crosslinked to collagen fibers by glutaraldehyde fixation and thereby retained during demineralization. (*B*) Top view transmission images revealing CBL's smooth mineralization front with adjacent osteoid (arrowheads) and numerous well‐defined cell lacunae (10nM Rap). (*C*) Similar images revealing DBL's ragged mineralization front without well‐defined osteoid (arrowheads), rough and poorly organized texture, and few well‐defined cell lacunae (10nM Rap). Boxes mark zoomed‐in regions shown in the correspondingly numbered panels. DBL = discontinuous bone‐like.

**Fig. 6:**
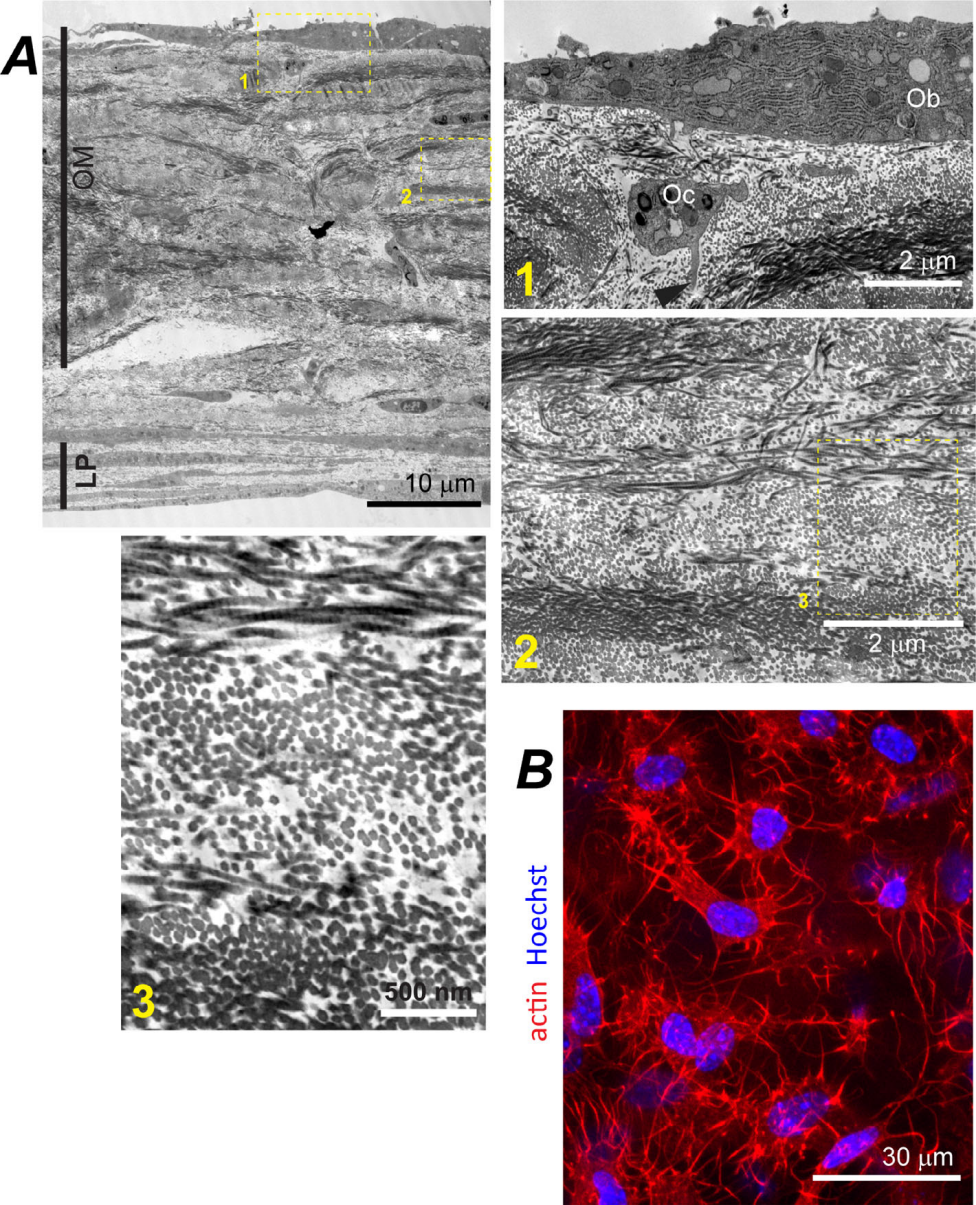
OM structure in primary PBC cultures. (*A*) Low resolution TEM image showing layering of OM and LP in a cross‐section of a primary PBC culture and higher resolution images of different OM regions marked by correspondingly numbered boxes (no βGP or Rap). Ob and Oc label osteoblast‐like and osteocyte‐like cells respectively; the arrowhead points to a process of the osteocyte‐like cell. (*B*) Confocal fluorescence image of a live primary PBC culture showing actin (red) cytoskeleton and nuclei (blue) of cells embedded inside an OM layer (no βGP or Rap). The image is an optical slice parallel to the layer. OM = osteoid‐mimicking.

**Fig. 7:**
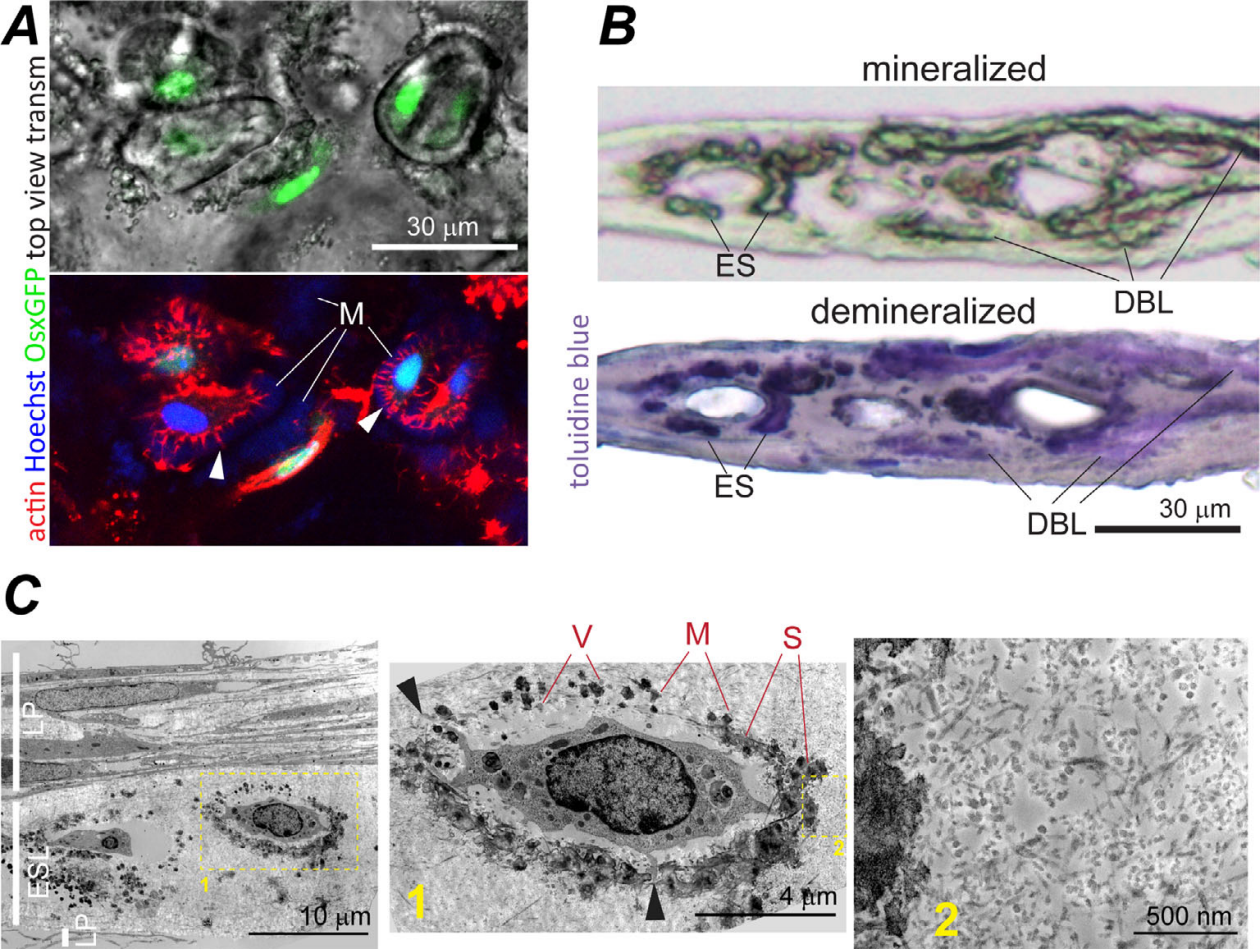
Eggshell‐like (ESL) structure in primary PBC cultures. (*A*) Top view transmission and confocal fluorescence images of ESL in live PBC culture expressing Cre::GFP in cells actively transcribing Sp7 (no βGP or Rap, c.f. Fig. 4*A,B*). In the top panel, GFP signal from cell nuclei is overlaid with the transmission image. The bottom panel image of the same area shows the same GFP signal (green), Sir‐Actin‐labeled actin cytoskeleton (red), and Hoechst‐labeled cell nuclei (blue). The fluorescence images are optical slices parallel to the ESL layer. Arrowheads point to cell processes in ovoidal lacunas. Letter M marks pericellular mineralized matrix (blue autofluorescence). (*B*) Transmission images of a culture cross‐section containing ESL before (top, 10×/0.3 NA objective) and after (bottom, 40×/0.55 NA objective) demineralization and toluidine blue staining (no βGP or Rap). ES points to mineralized pericellular matrix of an ovoidal lacuna. DBL marks mineralized DBL matrix. The sample was formaldehyde‐fixed before demineralization. (*C*) TEM images of ESL (no βGP or Rap). Low resolution TEM (left) shows LP and ESL layering in culture cross‐section. Zoomed‐in ESL regions (middle and right) marked by the correspondingly numbered boxes show ESL ultrastructure. The sample was glutaraldehyde‐fixed and not demineralized. V marks matrix vesicles. Very dark spots (M) around ovoidal lacunas are mineral crystals apparently nucleated by matrix vesicles. S marks staining smear around the mineral crystals apparently caused by negatively charged molecules (eg, mineral‐binding proteins) that bind the uranyl acetate stain. Similar smear appears in fixed bone and bone‐like samples (cf. Figs. 3*A*, 4*D*, 5*A*, and S7). Arrowheads point at cell processes.

Primary PBCs at 3–7 weeks past confluency revealed transparent patches of unmineralized structures, translucent patches of unmineralized structures, and opaque patches of mineralized structures in low magnification, top‐view transmission images (Fig. 1*A*). Higher magnification images in top view and cross‐sections revealed one or several distinct cell‐ECM structures (Fig. 1*B*) within each patch. Each structure had a distinct overall, ECM, and cell morphology and ultrastructure (Table 1, Figs. 3, 4, 5, 6, 7). Unmineralized, loosely packed (LP) structure with poorly organized, low‐density collagen matrix resembled periosteal cambium layer (Fig. 3). Continuous bone‐like (CBL) structure with mineralized, well‐organized, high‐density matrix was lamellar bone (Fig. 4). Discontinuous bone‐like (DBL) structure with mineralized yet less organized and medium‐density matrix resembled woven bone (Fig. 5). Unmineralized osteoid‐mimicking (OM) structure with well‐organized, medium‐density matrix had some semblance to nonmineralizing osteoid yet could be a cell culture artifact (Fig. 6). Eggshell‐like (ESL) structure with poorly organized, low‐density matrix and mineralized ovoid shells surrounding large cell lacunae had some semblance to embryonic cartilage yet also could be an artifact (Fig. 7). These structures were clearly distinguishable at 0mM and 1mM βGP but camouflaged and disrupted by rampant artificial ECM mineralization at 10mM βGP (Fig. 1*B*).

**Table 1:** Cell‐ECM Structures and Their Features Found in Primary Cell Cultures

				Cell types^a^			
Cell‐ECM structure	In vivo analog	Mineralization at ≤1mM βGP	Collagen matrix	Ob	Oc	Ch	Other
CBL (continuous bone‐like)	Lamellar bone	Continuous	Well‐organized, high density	+	+	−	−
DBL (discontinuous bone‐like)	Woven bone^b^	Discontinuous	Organized, medium density	+	+	−	+
OM (osteoid‐mimicking)	Undefined^c^	None	Well‐organized, medium density	+	?	−	−
LP (loosely packed)	Cambium^d^	None	Poorly organized, low density	−	−	−	+
ESL (eggshell‐like)	Undefined^e^	Pericellular	Poorly organized, low density	−	?	?	−

^a^ Ob, Oc, Ch, and “other” stand for osteoblast, osteocyte, chondrocyte, and other cell types, respectively. The presence of each cell type in the cell‐ECM structure is indicated by “+” (present), “−” (absent), or “?” (cells with some but not all expected features of the cell type are present).

^b^ Partial resemblance.

^c^ Has some semblance to nonmineralizing osteoid but might form only in vitro.

^d^ Only morphological and ultrastructural resemblance was examined.

^e^ Has some semblance to embryonic cartilage but might form only in vitro.

### LP cell‐ECM structure resembling cambium

**Fig. 8:**
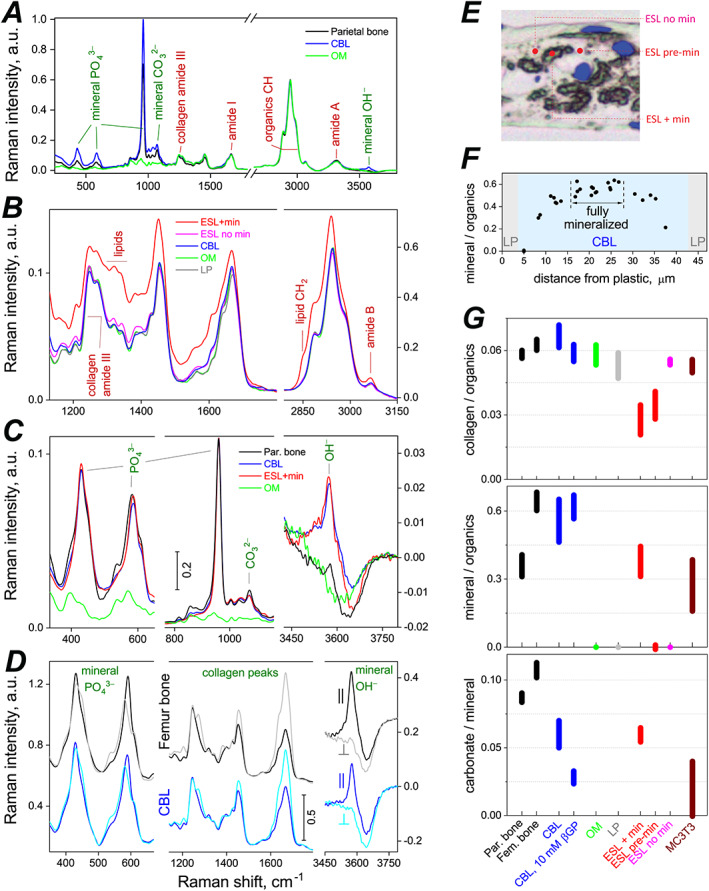
Confocal Raman microspectroscopy analysis of ECM composition and organization in compact bones and cell‐ECM structures grown in primary PBC cultures. Raman spectra of 8‐μm or 10‐μm cross‐sections of bones and cell‐ECM structures were measured within different structural features and away from cell lacunae with ~0.5 μm *x‐y* resolution. (*A*–*D*) Representative Raman spectra of fully mineralized compact bones and cell‐ECM structures grown without βGP. In *A*, *B*, and *D*, the spectra were scaled to have similar intensity of collagen amide III peaks. In *D*, femur and CBL spectra were also vertically offset. In *C*, parietal bone, CBL, and ESL spectra were scaled to match intensity of their 959 cm^‐1^ phosphate peaks whereas OM spectrum was scaled to match collagen amide III peaks. Panels *A*, *B*, and *D* show nonpolarized spectra. *D* shows polarized spectra with both excitation and emission polarizations set either parallel (||) or perpendicular (⊥) to collagen fibers. (*E*) Representative locations within ESL structure at which spectra of mineralized regions (ESL + min), unmineralized regions (ESL no min), and regions about to be mineralized (ESL pre‐min) were measured. (*F*) A profile of mineral phosphate to organic CH ratio across full thickness of a cell culture cross‐section that had a CBL structure (shaded in blue) in the middle. Fully mineralized region of the CBL is marked by a double arrow. Reduced mineralization on the sides indicates that mineral was deposited near the CBL center first, because mineralization at a given ECM spot takes ~3 weeks to proceed from nucleation to full level.^(^ ^40^ ^)^ Integral intensities of the 959 cm^‐1^ phosphate peak and organic CH peaks were used to calculate this ratio. (*G*) Ratios of integral intensities of collagen amide III to organic CH peaks (top), 959 cm^‐1^ mineral phosphate to organic CH (middle), and mineral carbonate to 959 cm^‐1^ mineral phosphate (bottom), representing ECM compositions in different cell‐ECM structures formed in culture and in vivo bones. In *G*, the bars show mean ± standard deviation; CBL bars represent an aggregate of similar data pooled from no βGP or Rap, 10nM Rap, and 1mM βGP cultures (Fig. S4*B*); CBL 10mM βGP bars represent data only from 10mM βGP cultures; all other bars represent data from no‐βGP/no‐Rap or 1mM βGP cultures (which had similar ECM compositions).

**Fig. 9:**
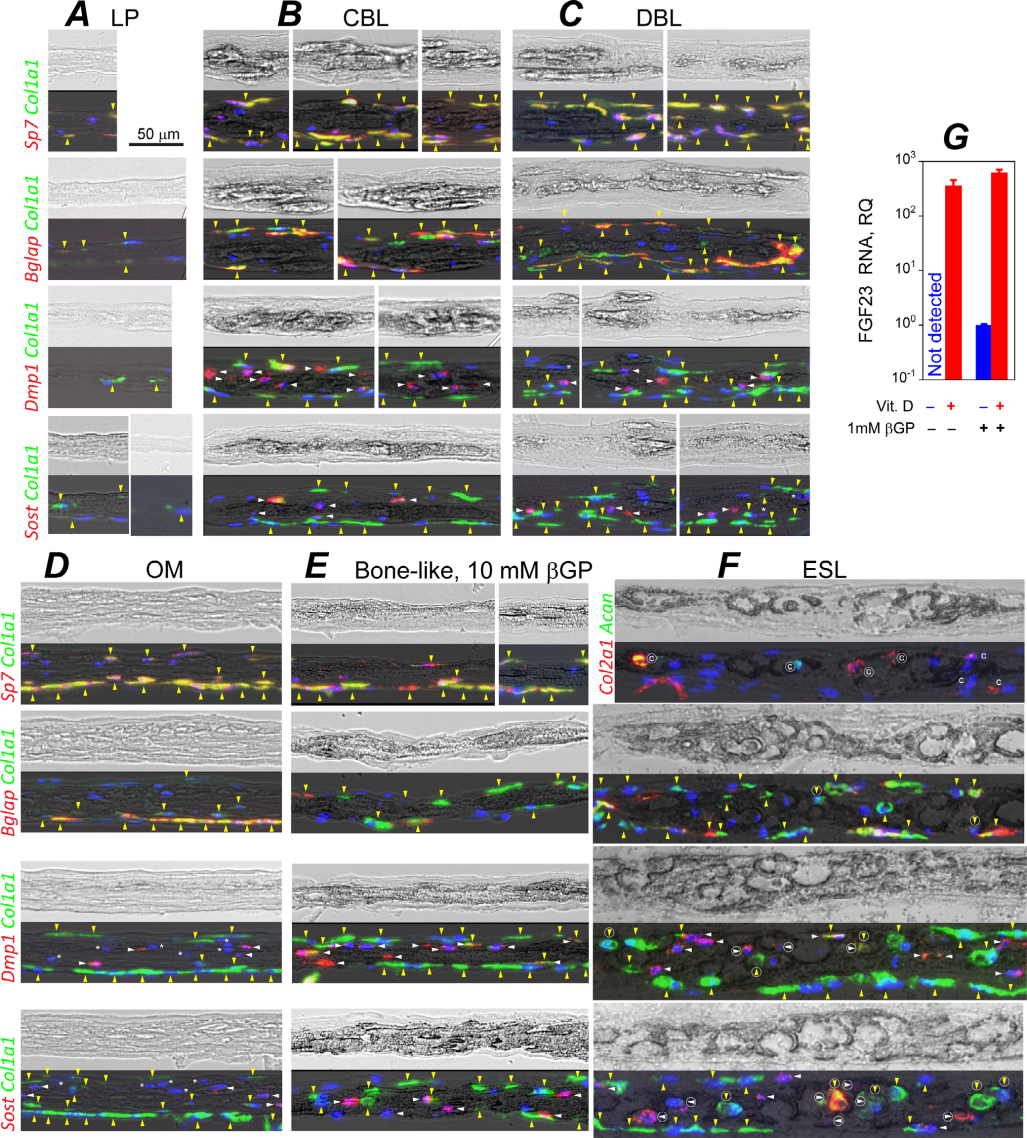
mRNA expression in LP (*A*), CBL (*B*), DBL (*C*), OM (*D*), artificially mineralized bone‐like (*E*), and ESL (*F*) cell‐ECM structures grown in primary PBC cultures. (*A*–*F*) Transmission images of PBC culture cross‐sections before demineralization (top) and the same images at reduced intensity overlaid with fluorescence signals from mRNA after staining (bottom). mRNA was fluorescently labeled by in situ hybridization with RNAScope™ probes for transcripts preferentially expressed in osteoblasts (*Bglap*, *Col1a1*, *Sp7*), osteocytes (*Dmp1*, *Sost*), and chondrocytes (*Acan*, *Col2a1*). All panels except *E* represent no‐βGP/no‐Rap or 1mM βGP cultures (in which each structure had similar appearance and mRNA expression). Vertical, yellow arrowheads mark cells expressing *Col1a1* (preosteoblasts, osteoblasts, and possibly fibroblasts). Horizontal, white arrowheads mark differentiating and mature osteocytes. Asterisks mark cells embedded in DBL and OM that do not express *Dmp1* or *Sost*. Letter C marks cells expressing *Col2a1* and/or *Acan*. Encircled arrowheads and letter C mark the corresponding cells inside mineralized ESL ovoids. We did not label signals which could be artifacts of sample preparation (eg, displaced cells or debris). (*G*) *Fgf23* mRNA transcription in mineralized primary PBC cultures in response to 2‐day treatment with 1α, 25‐dihydroxyvitamin D3. Error bars are standard deviations for *N* = 3 culture wells.

LP was the most ubiquitous cell‐ECM structure characterized by sparse, poorly organized bundles of thin collagen fibers and thin, spread‐out cells (Fig. 3*A*). Collagen fiber diameter distribution, volume fraction, and the extent of fiber fusion (Table 2, Fig. S2*B*) as well as ECM and cell morphology within LP resembled cambium layer of parietal bone periosteum (Fig. 3). LP was the only cell‐ECM structure within the transparent patches (up to ~40 μm in thickness and from tens of micrometers to millimeters in width), yet it also covered the bottom and sometimes top of other structures. LP was mechanically loose and prone to collapse due to very low volume fraction of collagen fibers (Table 2). Collagen and lipid fractions of LP ECM (measured by Raman microspectroscopy relative to all ECM organics) were close to parietal bone (Fig. 8*B,G*), suggesting that collagen was the dominant component of its ECM. Actin filaments in LP cells resembled myofibroblast stress fibers and were similar to those in parietal bone cambium (Fig. 3*B*). Fluorescent in situ mRNA hybridization showed that some of these cells simultaneously expressed *Col1a1* and *Sp7* (yellow arrowheads, Fig. 9*A*) but none had pronounced *Bglap*, *Dmp1*, or *Sost* expression, consistent with immature osteoblasts expected in cambium. Presence of cells not expressing *Col1a1* indicated a mixed cell population in LP (Fig. 9*A*).

**Table 2:** Collagen Extracellular Matrix Morphology in Parietal Bone and Cell‐ECM Structures in Culture

				Diameter of individual fibers	
	Matrix density	Volume fraction of all fibers	Fraction of fused fibers	Mean ± SE (nm)	Dispersion ± Err^a^ (nm)
Parietal bone	hdm	0.82–0.86	0.99–0.995	n.d.^b^	n.d.^b^
	ldm	0.55–0.69	0.84–0.94	20–55^b^	8.8 ± 0.4^b^
CBL	hdm	0.77–0.82	0.99–0.994	n.d.^b^	n.d.^b^
	ldm	0.45–0.52	0.87–0.92	20–60^b^	9.7 ± 0.5^b^
DBL^c^		0.17–0.33	0.19–0.80	35.4 ± 0.4	6.9 ± 0.3
OM		0.49–0.55	0.23–0.57	38.1 ± 0.3	7.7 ± 0.2
Parietal cambium		~0.06	~0.08	33.5 ± 0.3	6.4 ± 0.3
LP		0.04–0.06	0.02–0.07	34.7 ± 0.2	5.9 ± 0.15
ESL		0.06–0.10	<0.18	19.9 ± 0.2	5.3 ± 0.2

^a^ The dispersion (spread) of diameter distribution was calculated as the square root of the distribution variance. The standard error for the dispersion was calculated as described in Materials and Methods.

^b^ In parietal bone and CBL, collagen fiber morphology was analyzed separately within higher (*hdm*) and lower (*ldm*) density matrices. Individual fibers could be better distinguished in *ldm*, but extensive fiber fusion even in these structures resulted in low counts of individual fibers and some uncertainty in distinguishing thicker individual fibers from bundles of fused, thinner fibers. Therefore, a range instead of the mean fiber diameter is provided for *ldm*. In *hdm*, the diameter range and dispersion appeared to be similar to *ldm* but could not be reliably quantified (n.d. = not defined).

^c^ Only mineralizing sublayers of DBL were analyzed, see Results describing DBL.

### Cell‐ECM structures resembling bone

#### CBL structure

**Fig. 10:**
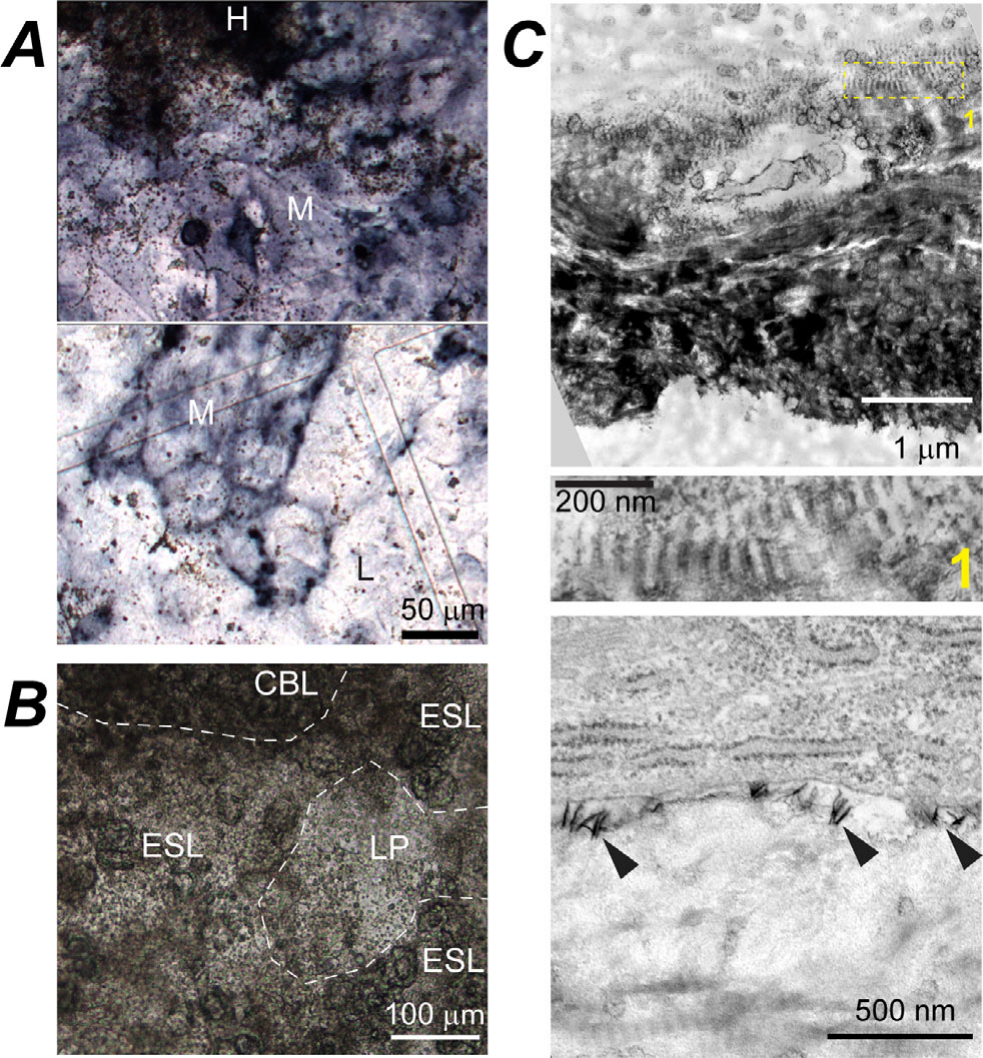
Artificial and normal mineralization induced by 10mM βGP in primary PBC cultures. (*A*) Top view, transmission images of a culture stained for ALP activity (dark blue/purple) at early mineralization stage (while mineralized nodules are small and sparse). Small brown dots are mineral granules. Dark, heavily stained region in the top panel (H) is a mineralized CBL nodule with densely packed mineral granules. Within regions with moderate ALP‐staining intensity (M), multiple mineral granules are present around well‐defined mineralized nodules (top panel) and fewer granules away from the nodules (bottom panel). Some mineral granules are also present in regions with low ALP‐staining intensity (L, bottom panel). (*B*) Top view, transmission image of a culture at intermediate mineralization stage showing numerous mineral granules within LP, CBL, and ESL structures, which begin to mask distinctive features of these structures and are about to merge into continuously mineralized plates (Fig. 1*B*) and then layer. (*C*) TEM images of a CBL cross‐section at an early mineralization stage, which was neither demineralized nor stained with uranyl acetate (mineral appears very dark). Zoomed‐in middle panel (box 1 in the top panel) shows normal mineralization within gap regions of collagen fibers, which matches the fiber D‐periodicity. The bottom panel shows abnormal deposition of needle‐like mineral crystals (arrowheads) at the surface of an early osteocyte‐like cell. ALP = alkaline phosphatase.

CBL was the structure most closely resembling perinatal parietal bone in features of its collagen fiber ultrastructure, overall ECM morphology, mineral and matrix composition, cells, and growth pattern. CBL was from tens to hundreds of micrometers wide. In cross‐section, CBL appeared as continuous, mineralized ECM, which was 15–45 μm thick and contained embedded cells like parietal bone (Fig. 1*B*). In top view (Figs. 1*B*, S5*A*,*B*, and S6*A*), CBL consisted of round or elongated, moderately opaque bone‐like spicules, which often interconnected into a network with trabeculae‐like appearance characteristic of early intramembranous ossification.^(^ ^48^ ^)^ In 5‐week cultures, CBL covered 1%–2% of total and 5%–10% of mineralized area, and its formation was not significantly affected by 1mM βGP or 10nM Rap (Fig. 1*C*). At 10mM βGP, CBL formed as well but could not be easily distinguished and quantified due to rampant mineralization of surrounding structures (Figs. 9*E* and 10*B*).

ECMs of CBL and parietal bone had similar collagen fiber density and appearance in TEM images, both within regions of higher (Fig. 4*D*) and lower (Fig. S7) matrix density (Table 1). They had similar imperfect lamellar fiber organization in polarized microscopy (Fig. 1*B*) and TEM (Fig. 4*D*) as well as similar range of individual fiber diameters, volume fraction of fibers, and degree of lateral fiber fusion (Table 2, Fig. S2*B*).

In fully mineralized CBL (Fig. 8*F*), mineral composition measured by Raman microspectroscopy was consistent with bone hydroxyapatite (Fig. 8*A*). Like in bone, CBL mineral had low hydroxyl content and contained carbonate ions (Fig. 8*C*,*D*), although the carbonate content was somewhat reduced (Fig. 8*G*, *p* < 0.001). Notably, 10mM βGP further reduced the carbonate content (Fig. 8*G*, *p* < 0.001). The mineral to organics ratio was close to that of similar, imperfectly organized lamellar bone in the femur cortex from 17‐week‐old mice and somewhat higher than in well‐organized lamellar parietal bone from 6.5‐week‐old mice (Fig. 8*G*). Mineral crystal orientation relative to collagen fibers was also close to the femur cortex regions that had similar organization of collagen fibers, as revealed by polarized Raman spectra (Fig. 8*D*). Note that these posterior mid‐diaphysis femur regions were used as in vivo control for mineral crystal orientation and mineral/organics ratio because their ECM was formed within similar timeframe as CBL (3–4 weeks before dissection), and had enough time to mineralize completely.^(^ ^40^ ^)^ This ECM was not altered by intracortical remodeling or cortical drift. In parietal bone such imperfectly organized lamellar ECM did form in young pups, but it was resorbed and replaced with well‐organized lamellar ECM before its mineralization was completed due to rapid outward growth of the skull.

CBL cell morphology, ultrastructure, and gene expression were similar to osteoblasts and osteocytes in perinatal parietal bone. The cells at CBL surface were thick, densely packed with the ER (Fig. 4*D*), and marked by high *Sp7*, *Col1a1*, and *Bglap* expression (yellow arrowheads, Fig. 9*B*), resembling mature osteoblasts. Embedded cells were smaller, had significantly less ER, formed an extended network of interconnected processes (Fig. 4*A*,*B,D*), and had low or no expression of *Col1a1* and *Bglap* yet high expression of *Dmp1* and *Sost* (white arrowheads, Fig. 9*B*), resembling differentiating and mature osteocytes. Upregulation of *Fgf23* expression in mineralized cultures after vitamin D treatment further supported full differentiation of cells into mature osteocytes (Fig. 9*G*).

CBL's growth pattern was also consistent with intramembranous ossification of perinatal calvaria. Time‐lapse imaging of cell‐ECM structures and fluorescent reporters of *Sp7* and *Bglap* expression (Figs. S5*B*,*C* and S6*A,B*), pulse fluorochrome labeling (Fig. 4*C*), and measurement of mineral density profiles (Fig. 8*F*) revealed the following four things. (i) After 1–2 weeks in culture (shortly post‐confluency), cells form clusters, become cuboid, and begin depositing ECM with high collagen density (Figs. S5*B* and S6*A*), resembling intramembranous ossification nidus.^(^ ^49^ ^)^ (ii) Subsequently (usually 2–3 weeks post‐confluency), cells expand the ECM laterally and transversally. Some cells become embedded and nucleate mineralization in the middle of the matrix (Figs. 4*C* and 8*F*), similar to mineralization induction in an osteocyte cell line.^(^ ^43^ ^)^ (iii) Further lateral expansion of CBL occurs through deposition and subsequent mineralization of osteoid (Figs. 5*B* and S5*A*,*B*), resembling appositional bone growth in vivo. (iv) The mineralizing clusters keep growing laterally and merge (if cluster density is high enough) forming network of CBL spicules with viable embedded cells (Fig. S5*B*). Overall, the cells remained viable and continued building CBL for at least 50–80 days in culture. Transversal growth of the spicules was restricted to less than ~45 μm, perhaps limited by the diffusion of oxygen and nutrients.

In CBL, ECM was lamellar and better organized than woven ECM of intramembranous ossification nidus in vivo. Instead, it resembled ECM formed by apposition on a bone surface, potentially because of the orienting effect of the culture dish plastic. In vivo, nidus cells lack such well‐defined support surface and are surrounded by preexisting connective tissue that affects nidus matrix deposition.^(^ ^48^ ^)^

#### DBL structure

DBL had several common features with parietal bone and CBL but different collagen organization and continuity of mineralized ECM (Figs. 1*B* and 5). In most cultures, DBL was the predominant mineralized cell‐ECM structure. The only exceptions were cultures that formed extensive eggshell‐like structure described in ESL structure Section below. DBL formation was significantly enhanced by 1mM βGP (Fig. 1*C*).

The main feature distinguishing DBL from CBL was interruptions of bone‐like structures by poorly organized, unmineralized sublayers, making DBL‐containing patches opaquer than CBL patches of similar thickness (Figs. 1*B* and 5*C*). ECM of the mineralized, plate‐like DBL sublayers had lower volume fraction of collagen fibers than CBL; its fibers were less fused and had smaller and more tightly distributed diameters (Table 1, Figs. 5A and S2*B*). This ECM lacked lamellar organization and was more heterogeneous, exhibiting much higher variation of the volume fraction of collagen fibers and of the fraction of fused fibers than all other cell‐ECM structures formed in culture (Table 2). Unmineralized sublayers within DBL had lower collagen fiber density than mineralized DBL sublayers yet higher than LP (Fig. 5*A*, middle and bottom layers of panels 1 and 2, respectively). DBL surface cells had *Sp7*, *Col1a1*, and *Bglap* transcription comparable to CBL osteoblasts (Fig. 9*B,C*). Most cells inside DBL were not completely surrounded by mineralized ECM (Fig. 5*A*), but their ultrastructure as well as *Dmp1* and *Sost* expression were often similar to CBL osteocytes (Fig. 9*B,C*). In the top view, DBL could be distinguished from CBL by the irregular/corrugated growing edge, lack of well‐defined mineralization front, and lack of well‐organized and clearly visible osteocyte lacunae (Figs. 1*B* and 5*B*,*C*). During early stages of formation, DBL often exhibited multiple events of bone nodule nucleation at the same location (Fig. S5*C*), apparently resulting in poorly organized, stacking layers of osteoblast‐like cells and matrix (Figs. 5*A* and 9*C*), whereas CBL exhibited a single nucleation and osteoblast‐like layers only on the top and bottom (Figs. 9*B* and S5*C*). Unlike CBL, DBL had some embedded cells with no *Dmp1* and *Sost* expression (white asterisks, Fig. 9*C*). These DBL features, however, do not necessarily mean nonphysiological mineralization by abnormal osteoblasts but may simply indicate disorganized, woven‐bone‐like nucleation of bone nodules that collide and overlap each other (eg, due to overcrowded nucleation of the nodules, Fig. S5*C*).

#### Other cell‐ECM structures

Aside from cambium‐like LP and bone‐like CBL and DBL, we observed two additional types of cell‐ECM structures that had features not observed in perinatal parietal bone in vivo. These features were related to altered osteoblastic differentiation and function and might be cell culture artifacts.

#### OM structure

OM resembled osteoid in appearance, but unlike CBL's osteoid, it did not mineralize at ≤1mM βGP and had distinct morphology, ultrastructure, and gene expression. It covered the largest fraction of surface area in cultures without βGP or Rap and with 1mM βGP (Fig. 1*C*). Its areal fraction was significantly reduced by Rap.

OM frequently occurred in large unmineralized translucent patches, reaching up to millimeters in width (Fig. 1*A*), which typically consisted of thin (~10 μm) LP overlaid by a thicker (up to ~45 μm) OM (Figs. 1*B* and 6*A*). OM's ECM had lamellar organization as well as collagen and lipid fraction like in CBL (Figs. 1*B* and 8*A,B,G*). However, unlike CBL, OM had an even thickness over large areas without spicules or interconnected trabeculae. OM's collagen fiber density, diameter distribution, and fraction of fused fibers were intermediate between CBL and LP and distinct from mineralized layers of DBL (Table 2, Fig. S2*B*). Cells at the OM surface resembled cells at the CBL surface in morphology, dense ER packing, and high expression of *Sp7*, *Col1a1*, and *Bglap* (Figs. 9*D* and 6*A*). Like in CBL, cells embedded in OM had an extensive network of interconnected processes (Fig. 6*B*), but unlike CBL and DBL, a large fraction of these cells did not express *Dmp1* and *Sost* (white asterisks, Fig. 9*D*). Apparently, osteoblasts forming OM had altered maturation and differentiation into osteocytes, resulting in a distinct ECM structure and lack of mineralization. Mineralized patches sometimes appeared within OM areas in top view, but cross‐sections revealed them to be DBL overlaying OM (Fig. S5*D*).

#### ESL structure

ESL was distinguished by mineralized ovoids, did not resemble parietal bone structures, appeared to be produced by a distinct subpopulation of cells, and had ECM, mineral, and cells with mixed features of bone and cartilage. Each ovoid morphologically resembled an eggshell both in the top‐view and in cross‐section (Figs. 1*B* and 7). The ovoid shells were often discontinuous, likely because of focal mineralization (Fig. 7*B,C*). ESL appeared in PBC cultures 3–7 days after confluency. Ovoid clusters were tens to hundreds of micrometers wide and 30–60 μm thick. The areal fraction of ESL varied from undetectable in some cell preparations (Fig. 1*C*) to ~80% of mineralized area in other preparations (not shown), suggesting ESL formation by a specific, highly variable subset of cells (see Fig. S8 for discussion of their origin).

ECM outside mineralized ovoid shells had collagen fibers with similar density to LP but much smaller diameter (Table 2, Fig. 7*C*), resembling the 18‐nm fibers abundant in embryonic hyaline cartilage^(^ ^50^, ^51^ ^)^ rather than bone or cambium fibers. This ECM, however, had very faint toluidine blue staining, suggesting low content of sulfated glycosaminoglycan chains normally abundant in cartilage (much fainter even than in adjacent DBL structures after demineralization, Fig. 7*B*). Its collagen:organics ratio was similar to bone (Fig. 8*B*,*G*), consistent with the low proteoglycan content.

Mineral within the ovoid shells was hydroxyapatite with carbonate and hydroxyl contents similar to CBL (Fig. 8*E,C,G*). Unlike CBL and DBL, ESL mineralization localized to pericellular matrix only (except in 10mM βGP) and appeared to be nucleated by extracellular vesicles (Fig. 7 *C*), consistent with the higher lipid content and lower collagen:organics and mineral:organics ratios evaluated from Raman spectra (Fig. 8*B*,*E,G*). Dark (compared to the adjacent DBL) toluidine blue staining of mineralized ovoid shell regions indicated increased content of negatively charged molecules (eg, mineral‐binding proteins) localized within these regions (Fig. 7*B*,*C*).

Each ovoid appeared to be formed by the cell it enclosed. Rounded morphology of the cell lacunae and nuclei resembled chondrocytes, yet the cells lacked extended ER and had osteocyte‐like morphology with numerous processes protruding into pericellular matrix. Unlike bone and CBL, the processes were short and not connected into extended network (Fig. 7*A*). Some of the cells residing in ESL clusters (inside and outside of well‐defined ovoids) had high expression of chondrocyte markers *Col2a1* and *Acan*, whereas other cells expressed osteoblast and osteocyte markers *Col1a1*, *Bglap*, *Dmp1*, and *Sost* (Fig. 9*F*). The presence of the latter cells within chondrocyte‐like lacunae (encircled arrows, Fig. 9*F*) may indicate chondrocyte to osteoblast/osteocyte transdifferentiation.

### Artificial ECM mineralization at 10mM βGP

At 10mM βGP, the same LP, CBL, DBL, OM, and ESL cell‐ECM structures were distinguishable initially (Fig. 10*B*) but disrupted and camouflaged later by rampant mineralization upon inorganic phosphate release from βGP by alkaline phosphatases (ALPs). At early stages, we observed formation of mineral granules within and next to areas of high ALP activity (Fig. 10*A*). The granules formed even within LP and OM layers that did not mineralize without βGP or at 1mM βGP. Raman microspectroscopy of areas with sparse collagen fibers revealed the granules to be hydroxyapatite with no preferred crystal orientation, which were likely formed by nonspecific calcium phosphate precipitation.^(^ ^42^ ^)^ This granular mineralization occurred irrespective of ECM morphology, eventually filling all culture regions except for extended LP areas not overlaying other structures (Figs. 1*A,B* and 10*A,B*). The rampant mineralization altered gene expression (cell differentiation) and appeared to kill some cells (large regions of fully mineralized culture contained numerous empty cell lacunae). Additionally, 10mM βGP could alter osteoblast differentiation directly as it delayed mineralization in subclone 4 of MC3T3‐E1 cell line (no mineral formed in 5 weeks, Fig. S4*B*). In CBL at 10mM βGP, the mineral carbonate content was reduced but overall mineral and collagen contents, mineral orientation, and mineral deposition within gap regions of collagen fibers were normal (Figs. 8*G* and 10*C*).

Another curious artifact of high βGP concentration was abnormal nucleation of needle‐like mineral crystals at cell surfaces (Fig. 10*C*), which was likely induced by cell surface phosphatases hydrolyzing βGP and was not observed without 10mM βGP. The contribution of this process to overall mineralization and its effect on cellular function were difficult to evaluate, because these crystals were detectable only by electron microscopy of fixed samples. We did not observe mineralization caused by calcium release from organelles of dying cells and subsequent calcium phosphate precipitation in cell lacunae and pericellular matrix, which was previously reported as another artifact at high βGP.^(^ ^27^, ^29^, ^52^ ^)^

## Discussion

It is known that outcomes of mineralization experiments vary with cell source, preparation, seeding density, culture media, and other conditions.^(^ ^4^, ^8^, ^12^, ^13^, ^14^, ^15^, ^19^, ^25^, ^28^, ^53^, ^54^ ^)^ However, the diversity of bone‐like and other cell‐ECM structures, their physiological relevance, and the role of cell culture conditions in this diversity have not been fully appreciated. Our study suggests that full understanding of mineralized cultures requires: (i) identifying structures produced by primary osteoblasts at low concentrations (≤1mM) of βGP, a supplement not required for bone‐like mineralization; (ii) identifying effects of cell culture conditions on these structures and their mineralization; and (iii) identifying potential artifacts caused by artificial (nonphysiological) mineralization at more commonly used higher βGP concentrations.

### Bona fide bone formation by cultured primary cells

Probably the most important lesson from this study is that primary mouse PBCs cultured on a 2D substrate can progress through differentiation from osteoblast precursors to mature osteocytes and form CBL with many features of lamellar bone. ECM and mineral composition (Fig. 8*G*), growth pattern (Figs. 4*C*, S5*B,C*, and S6), ECM and cell morphology, ECM and cell ultrastructure (Figs. 4*A,B,D* and S7), and cell phenotypes (gene expression, Fig. 9*B*) in CBL are similar to perinatal parietal bone. Top view transmission images of trabeculae‐like structures resembling CBL reported previously in rat calvarial cell cultures at 2mM βGP^(^ ^29^, ^54^ ^)^ suggest that CBL may form in other experimental settings as well.

PBCs also form DBL with several features of bone but poorly organized bone‐like ECM and intrusions of non‐bone‐like ECM and cells (Figs. 5*A,C* and 9*C*). Time‐lapse tracking of formation (Fig. S5*B*,*C*) and gene expression imaging (Fig. 9*B*,*C*) suggest that the non‐bone‐like ECM and cell intrusions into DBL may be entrapped upon nucleation and growth of overlaying bone nodules rather than caused by disrupted osteoblast differentiation. Thus, DBL might be related to in vivo woven bone, and distinguishing CBL and DBL might not be essential for evaluating osteoblastic differentiation. *Fgf23* expression in response to active vitamin D (Fig. 9*G*) suggests that osteocytes embedded in CBL and DBL are functional. Altogether, multiple techniques suggest that CBL is bona fide bone, and DBL might be a cell culture proxy of bone.

### Diverse cell‐ECM structures and cell differentiation outcomes

Another key lesson is the observed diversity of mineralized and unmineralized cell‐ECM structures in culture. In addition to CBL and DBL, PBC cultures formed mineralized ESL structures that do not represent the osteogenic potential of the cells. ESL has ECM, mineralization pattern, cellular morphology, and gene expression indicative of mixed chondrogenic and osteogenic cell differentiation and possibly chondrocyte to osteoblast transdifferentiation (Figs. 7, 8, 9). Partial chondrogenic differentiation of PBCs might be an artifact of in vitro culture, shifting the cells from intramembranous ossification toward cartilaginous mineralization, eg, due to transient incubation at 5% oxygen used in this work. Still, ESLs might be of interest for future studies given the reports of chondrocyte‐like osteoprogenitor cells^(^ ^55^, ^56^ ^)^ and chondrocyte‐to‐osteoblast trans differentiation in vivo.^(^ ^57^, ^58^, ^59^, ^60^ ^)^

Two other types of abundant cell‐ECM structures in PBC cultures are unmineralized and exhibit only artificial mineralization at high βGP concentrations. OM structure morphologically resembles osteoid, but its growth pattern, size, shape, and collagen density (Figs. 6 and 8, Table 2) are distinct from a cell culture version of the physiological osteoid (Figs. 5*B* and S5*A*). Gene expression in most of the cells embedded in OM indicates deficient osteocytic differentiation (Fig. 9*D*), but we do not know the cause of this deficiency and whether it is related to nonmineralizing osteoid observed in bone pathologies.^(^ ^1^ ^)^ LP structures growing alone or coating other structures (Fig. 3) are produced by cells not differentiated into mature osteoblasts (Fig. 5). Morphologically and ultrastructurally, LP resembles the cambium layer of parietal or femoral periosteum, but we do not know whether it is a useful cambium model (Fig. 3). Detailed analysis of OM and LP cell‐ECM structures was beyond the scope of this work but might be of interest for future studies.

Distinguishing mineralized CBL, DBL, and ESL as well as unmineralized LP and OM structures may thus be beneficial for the analysis of normal and pathological osteoblast differentiation in culture. However, imaging techniques traditionally used in cell culture mineralization assays, particularly the approaches based on mineral staining, can hardly discriminate between these structures and may be misleading. Gene expression profile of the whole culture may also be insufficient to indicate normal osteoblast differentiation because cells in non‐bone‐like OM and ESL structures express mature osteoblast and osteocyte genes (Fig. 9*D*,*F*). In contrast, all these structures are easily distinguishable in live or fixed, unstained cultures under a conventional cell culture microscope with moderate magnification and optimal settings as described in Materials and Methods.

### Cell‐ECM structures formed by MC3T3‐E1 cells

We also found that MC3T3‐E1 subclone 4 cells, which is a popular model for studying osteoblast biology in culture, do form mineralized structures with some resemblance to CBL yet exhibit deficient osteocytic differentiation (Figs. 2, S3, and S4*A*,*C*,*D*). Osteocyte‐like cells in these structures are much sparser than in CBL or DBL structures formed by primary PBCs (Figs. 4*A* and S4*C*). MC3T3‐E1 subclone 4 cells have low expression of *Alpl*, *Dmp1, Mepe*, and *Sost* (Fig. 2) and form fewer and shorter processes not connected into extended networks when embedded in mineralized matrix (Fig. S4*C*). Nonetheless, the mineralized structures and expression of osteoblast markers (Fig. 2) are consistent with using this cell line for recapitulating some features of osteoblasts.^(^ ^61^ ^)^ MC3T3‐E1 cells also provide a useful model for investigating how osteoblasts produce, fold, and traffic procollagen.^(^ ^34^, ^62^, ^63^ ^)^ Still, one needs to keep in mind that collagen matrix reported for even better mineralizing subclone 14 of MC3T3‐E1 cells^(^ ^64^ ^)^ is less dense and more disorganized than matrix of CBL or bone in vivo (Figs. 6*D* and S7).

### Culture conditions affecting osteogenic differentiation

CBL and DBL areal fractions depend on the culture medium and other conditions (see, eg, Fig. 1*C*). Given known serum effects on shifting progenitor differentiation toward osteoblasts, chondrocytes, or adipocytes, our experiments were performed with serum pretested to support differentiation of mouse BMSCs into osteoblasts. Additionally, our media for primary cells were always supplemented with 100μM Asc2P because ascorbic acid is essential for maintaining collagen synthesis and therefore normal osteoblast function.^(^ ^31^, ^33^ ^)^ MC3T3‐E1 cells were differentiated in the same media supplemented with Asc2P but had to be maintained in undifferentiated state using media without Asc2P or ascorbic acid, which could affect their differentiation capacity.

#### Cell expansion

Because osteoblast environment (surrounding cells, ECM, and culture dish surface) affects osteoblast differentiation^(^ ^53^ ^)^ and because mature osteoblasts do not proliferate, the approach to expanding cells to confluency might affect subsequent cell differentiation. Cell expansion in growth media without added Asc2P or ascorbic acid is common, yet it may affect the capacity of the cells to undergo subsequent osteogenic differentiation because of severe disruption of osteoblast ER by accumulating procollagen.^(^ ^34^, ^65^ ^)^ In our experience, expanding primary cells in the growth medium supplemented with 100μM Asc2P in a regular incubator with atmospheric (~20%) oxygen ensured efficient CBL and DBL formation when the cells were seeded at ~10,000/cm^2^ or higher density. CBL and DBL formation at lower seeding density was inconsistent, apparently because 20% O_2_ inhibits cell proliferation.^(^ ^35^, ^36^ ^)^ An alternative approach described in the present study is seeding fewer cells at ~2000/cm^2^, expanding to confluency at 5% O_2_ (promoting proliferation without affecting differentiation potential^(^ ^35^, ^66^ ^)^) and differentiating at ~20% O_2_. Interestingly, cultures expanded in growth medium with 100μM Asc2P and subsequently incubated at 5% O_2_ for a month deposited thick cell‐ECM layer and had similar expression of *Ibsp*, *Bglap*, *Dmp1*, *Mepe*, *Phex*, and *Sost* to cultures transferred to 20% O_2_ at confluency, but they did not mineralize in the absence of βGP. This observation is consistent with a previous study.^(^ ^67^ ^)^

#### Osteogenic differentiation

Growth medium supplemented with 100μM Asc2P at 20% O_2_ is sufficient for CBL and DBL formation, but medium supplementation with Rap or 1mM βGP post‐confluency enhances nucleation of mineralization. Rap may affect differentiation by inhibiting cell proliferation.^(^ ^68^ ^)^ It accelerates appearance of mineralized nodules, distributes them more evenly across the culture well (Fig. 1*A*), and makes CBL more optically transparent and easier to study (Fig. S5*A*). Yet we do not know whether it creates conditions deviating from physiological bone formation.

Treatment with 1mM βGP accelerates nucleation and increases overall mineralization by enhancing DBL formation (Fig. 1*C*), consistent with previous reports of osteogenic differentiation stimulation with βGP.^(^ ^25^ ^)^ Some nonspecific calcium phosphate precipitation occurs already at 1mM βGP, but it does not significantly affect identification and overall assessment of different cell‐ECM structures. A drawback of this treatment is that it increases the relative contribution of DBL, yet it does not appear to affect the overall CBL formation (Fig. 1*C*).

### Effects of high βGP concentration on assessing cellular osteogenic potential

In contrast to low (≤1mM) concentration, more commonly used 10mM βGP (i) causes rampant artificial mineralization of mouse PBC cultures that impedes visualization of CBL and other structures (Fig. 10*B*), and (ii) causes cell death in these cultures (numerous empty lacunae in extensively mineralized regions), as described before for mouse and rat calvarial cell cultures.^(^ ^29^, ^54^ ^)^ These effects present a problem for assessing osteogenic potential of the cells. In PBC cultures, phosphate release from βGP by ALPs at 10mM βGP causes uncontrolled calcium phosphate precipitation and hydroxyapatite deposition that resembles calcification rather than osteogenesis (Fig. 10*A,B*), as reported.^(^ ^52^, ^69^ ^)^ This artificial mineralization engulfs all cell‐ECM structures (including LP, OM, and ESL), masks their distinguishing morphological features (Fig. 10*B*), and affects viability of the cells (potentially by blocking their access to nutrients and oxygen). Even within CBL, it causes nucleation of abnormal mineral crystals (Fig. 10*C*) and reduces carbonate content of the mineral (Fig. 8*G*).^(^ ^25^ ^)^ Therefore, interpretation of mineralization at 10mM βGP (eg, based on Alizarin red or von Kossa staining) as osteogenic differentiation of the cells rather than just ALP expression does not seem justified, as suggested before.^(^ ^29^ ^)^

Another detrimental effect of 10mM βGP is inhibition of proper osteogenic differentiation of both mouse PBCs (Fig. 2, *Bglap*) and MC3T3‐E1s (Fig. S4*B*). In MC3T3‐E1 (subclone 4) cells expressing much less *Alpl* (and thereby ALP, Fig. 2) than PBCs, 10mM βGP significantly delays mineralization (opposite to 1mM βGP, Fig. S4*B*), suggesting that 10mM βGP may also affect the cells and inhibit osteogenic differentiation directly.

## Conclusions

Primary parietal bone cultures can form CBL cell‐ECM structure, which resembles in vivo intramembranous bone formation in multiple structural and functional features.

Cell cultures also form other mineralized and unmineralized structures, which contain misdifferentiated cells and lack at least some key bone features. These structures may model pathological rather than normal osteogenesis and can be more abundant than CBL.

Assays of osteogenic potential of mouse PBCs by mineral detection without additional information (eg, CBL/DBL identification) are prone to artifacts due to mineralization of non‐bone‐like structures, particularly at 10mM βGP concentration commonly used in such assays. Gene expression profile of the whole culture may be insufficient for evaluating osteogenicity either due to expression of osteoblast and osteocyte genes within the non‐bone‐like structures.

Evaluation of CBL (and potentially DBL) formed without βGP or at low βGP concentrations (~1mM) is a more reliable approach to assessing osteogenic differentiation potential of mouse PBCs, which can be implemented with a simple cell culture microscope as described in Materials and Methods and Fig. 1*B*.

Optical transparency (particularly high in rapamycin‐treated cultures) and osteocyte survival up to several weeks make CBL a good in vitro model for mechanistic studies of bone biology, by, eg, in situ visualization of gene expression, microspectroscopic analysis of mineral and ECM, or high‐resolution imaging of ECM and cells outside and inside mineralizing matrix in live cultures (eg, Fig. 6*A*).

## Author Contributions

**Edward L Mertz:** Conceptualization; data curation; formal analysis; investigation; methodology; software; validation; visualization; writing – original draft; writing – review and editing. **Elena Makareeva:** Conceptualization; data curation; formal analysis; investigation; methodology; project administration; resources; supervision; validation; visualization; writing – original draft; writing – review and editing. **Lynn Mirigan:** Investigation; writing – review and editing. **Sergey Leikin:** Conceptualization; data curation; formal analysis; funding acquisition; investigation; methodology; supervision; validation; visualization; writing – original draft; writing – review and editing.

## Conflicts of Interest

All authors declare having no conflicts of interest and not being subjects of any institutional investigations.

### Peer Review

The peer review history for this article is available at https://publons.com/publon/10.1002/jbm4.10701.

## Supporting information


**Appendix S1.** Supporting information
Figs. S1–S8

## Data Availability

Data available upon request from the authors.
