# Supplementary material for: Bone Formation in 2D Culture of Primary Cells
Source: JBMR Plus. 2022 Dec 13;7(1):e10701. doi: 10.1002/jbm4.10701 (PMC9850442; doi:10.1002/jbm4.10701)
Supplement: Supplementary file 1 — Appendix S1. Supporting information Figs. S1–S8 [file JBM4-7-e10701-s001.pdf]

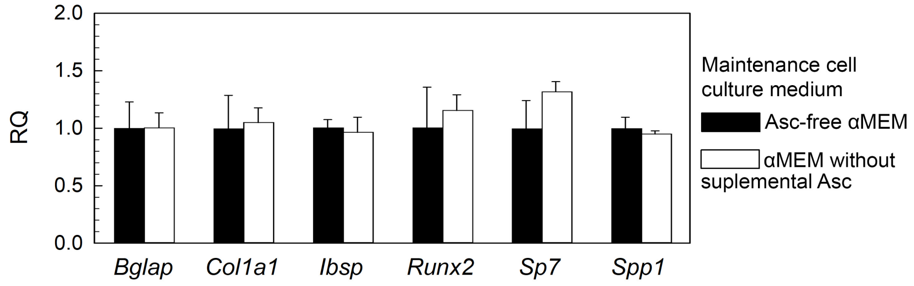

Supplemental Figure S1. Relative expression of key osteoblast differentiation markers in MC3T3-E1 subclone 4 cells maintained in ascorbic acid (Asc) free αMEM (A10490, ThermoFisher, recommended by ATCC) vs. regular αMEM (32571, ThermoFisher) without supplemental Asc. This figure demonstrates that the cells remain in the same undifferentiated state and can be maintained in either of the two media. The regular αMEM does not cause unintended osteoblastic differentiation of the cells without supplemental Asc (or Asc2P, long lasting form of Asc) because the Asc contained in it is inactivated by the time the media is received from the manufacturer (Refs. 33, 37-39 of main text). mRNA was quantified by qPCR and normalized to housekeeping genes as described in Fig. 2. The relative quantity (RQ) was measured relative to the average value in the Asc-free αMEM. The normalization reference for gene expression in Asc-free αMEM was measured by seeding the cells at the same density as for the Supp. Fig. S3 differentiation experiment and maintaining in Asc-free αMEM for 2 days (with a medium change 24 h after seeding). Expression of the same genes in cells maintained in regular αMEM represents the first time point in Fig. 2 differentiation experiment. This time point was measured 2 days after seeding the cells in αMEM without supplemental Asc2P (with a medium change 24 h after seeding). Note that differentiation of the cells is induced by 100 µM of Asc2P (Fig. 2 and Supp. Fig. S3), which begins only after cells reach confluence, clear misfolded procollagen from the cell ER and normalize Asc/Asc2P-dependent procollagen biosynthesis (~ 2 days after adding Asc2P, see Ref. 34 of main text). This differentiation is accompanied by ~ 100-fold increase (p<0.001) in the expression of *Bglap* and *Ibsp* by day 8 after seeding (Fig. 2 and Supp. Fig. S3).

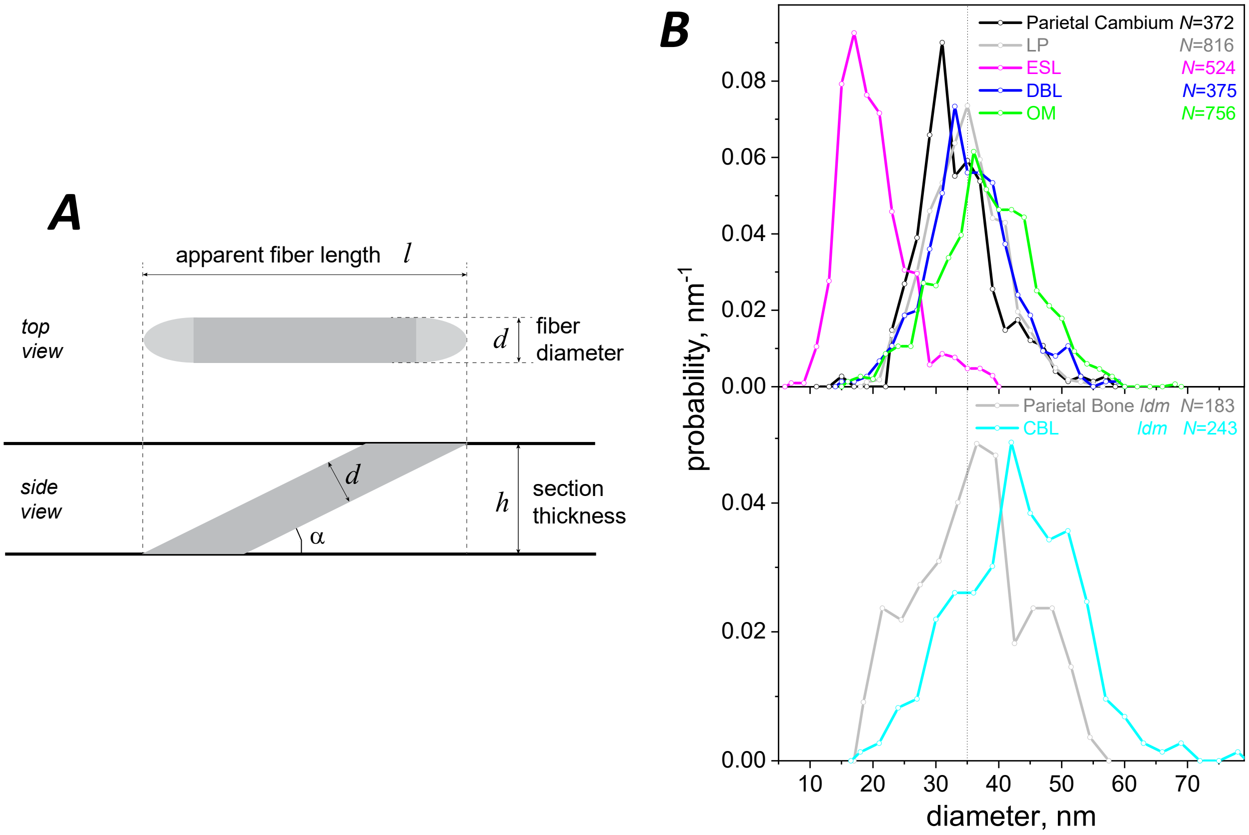

Supplemental Figure S2. Collagen fiber morphology analysis in TEM images. (*A*) Schematic representation of fiber volume fraction calculation within areas of tilted fibers. Assuming that fibers are long, straight cylinders, the fraction of the section volume occupied by each fiber is given by

$$v = \pi d^2/(4A \sin\alpha),$$

where *d* is the fiber diameter, *A* is the analyzed section area, and *α* is the fiber tilt angle relative to the section plane. The value of sin*α* can be calculated from

$$l = h/\tan\alpha + d/\sin\alpha,$$

where *h* is the section thickness and *l* is the apparent fiber length in the top view, yielding

$$v = \frac{\pi d^2}{4A}\frac{h\sqrt{h^2-d^2+l^2}-ld}{h^2-d^2}.$$

The total fiber volume fraction is the sum of the fractions occupied by each individual fiber in the analyzed area.

(*B*) Histograms of diameter distributions of individual (unfused) fibers in cultured cell-ECM structures, parietal bone, and parietal cambium (bone and cambium from a 3-day-old mouse). *N* is the number of analyzed fibers. ESL fibers are much thinner and more uniform in diameter than the fibers in all other structures (p<0.001). Diameter distributions of all but ESL structures are qualitatively similar to bone and cambium and to each other. Distributions of fiber diameters in LP, DBL and cambium are indistinguishable within the measurement uncertainty and somewhat narrower than in CBL and parietal bone (p<0.01). OM distribution is slightly wider and slightly shifted (by ~ 5 nm) towards larger diameters compared to LP, DBL, and cambium (p<0.01). The difference between CBL and parietal bone distributions for lower density matrices (*ldm*, analyzed here and shown in Supplemental Fig. S7) is not statistically significant and might not be real, because high fiber density and high extent of fiber fusion (Table 1) complicate identification and quantification of unfused fibers in CBL and parietal bone.

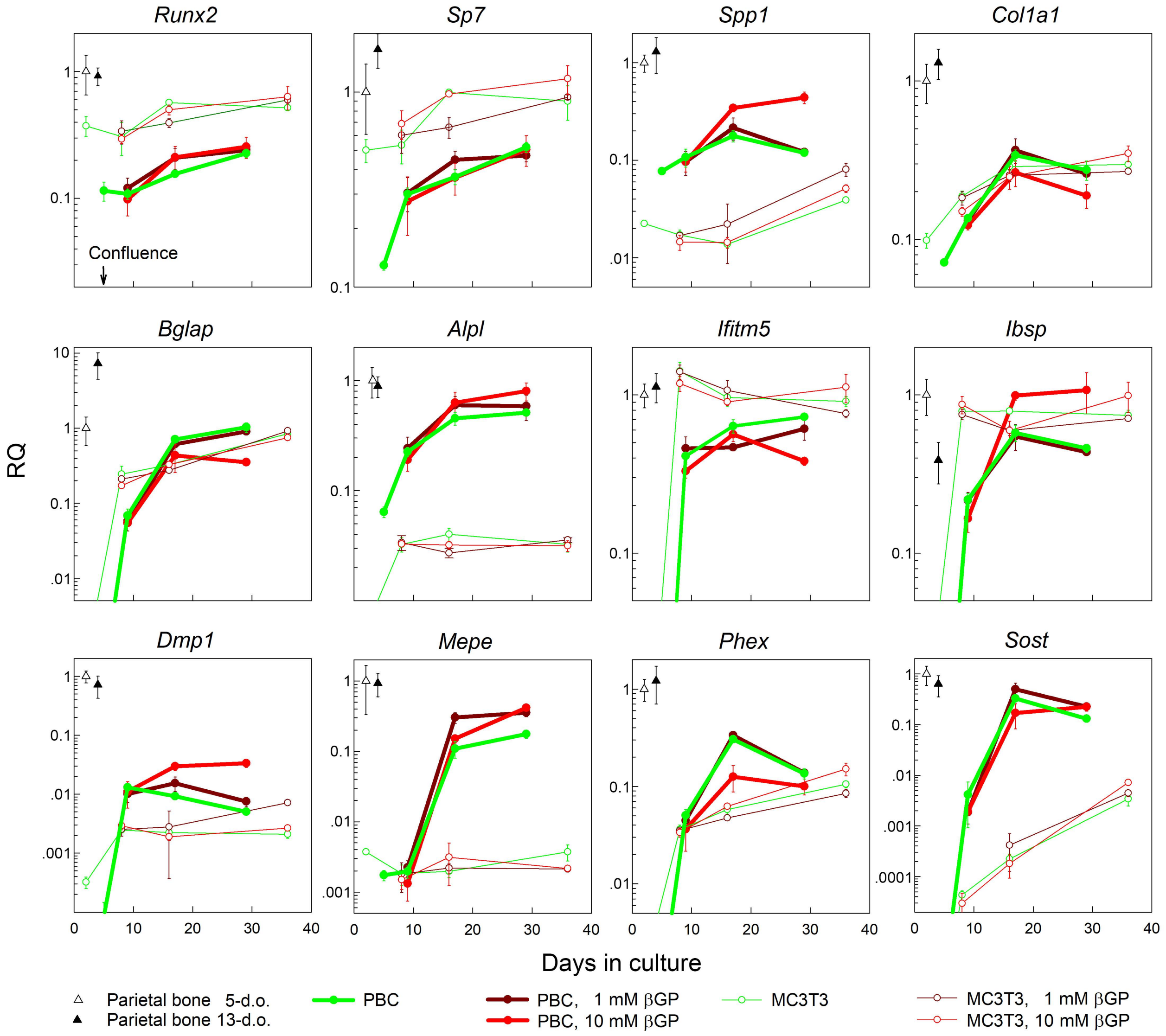

Supplemental Figure S3. Reproducibility of PBC and MC3T3-E1 subclone 4 cell differentiation in culture. This experiment repeats Fig. 2 using a different preparation of PBCs and a different stock of MC3T3-E1 cells, except the latter cells were maintained and seeded in Asc-free αMEM rather than regular αMEM without Asc or Asc2P supplementation (see Supp. Fig. S1). The error bars represent standard deviations for *N*=3 replicates. To ensure identical culture conditions (e.g., the same cell seeding density), the replicates are different wells seeded from the same suspension of cells pooled together from multiple animals as in the Fig. 2 experiment. All relative quantities (RQs) were calculated as described in Fig. 2 using the same housekeeping genes and normalization to the average values in parietal bones from 5-day-old mice. PBCs were seeded in αMEM supplemented with 100 µM Asc2P as in Fig. 2. MC3T3-E1 cells were maintained and seeded in Asc-free αMEM as described in Supp. Fig. S1. The first time point in MC3T3-E1 cultures was measured 2 days after seeding in Asc-free αMEM (Supp. Fig. S1). The other time points in these cultures were measured after differentiation initiated by replacing the medium to regular αMEM supplemented with 100 µM Asc2P 24 h after seeding. The results of this experiment are essentially identical to Fig. 2 measured several years earlier.

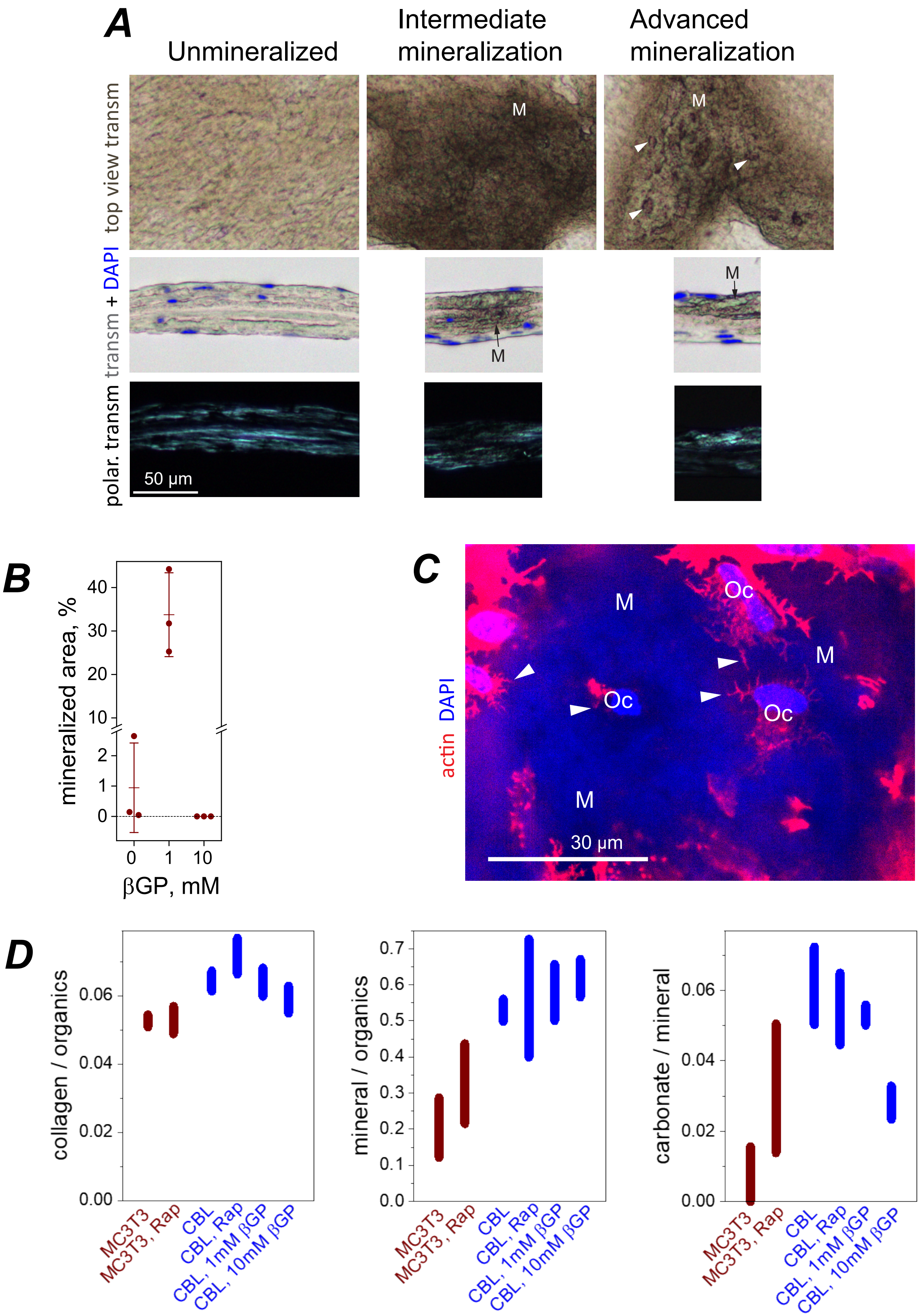
A
Unmineralized
Intermediate mineralization
Advanced mineralization
top view transm
transm + DAPI
polar. transm
M
50 µm
B
mineralized area, %
βGP, mM
C
actin DAPI
M
Oc
30 µm
D
collagen / organics
mineral / organics
carbonate / mineral
MC3T3
MC3T3, Rap
CBL
CBL, Rap
CBL, 1mM βGP
CBL, 10mM βGP

Supplemental Figure S4. Cell-ECM structures in cultures of MC3T3-E1 cells. (*A*) Top view and cross-section images of unmineralized structures and structures at intermediate and advanced mineralization stages (all measured at 5.5 weeks after plating in cultures without βGP or Rap). At these and other studied conditions, unmineralized, collagen-rich nodules (left panels) deposited disconnected mineral particles (M, middle panels). These particles subsequently merged into mineralized continuum (M, right panels), making mineralized matrix more transparent and cell lacunas (arrowheads) inside the matrix visible. The resulting mineralized nodules acquired semblance to CBL and parietal bone (cf. Fig. 1B). Color balance and intensity of the bright-field transmission images were corrected as described in Methods. (*B*) Effect of βGP on mineralization in MC3T3-E1 cultures 5.5 weeks after plating. Each point represents a separate well within the same plate. (*C*) Confocal fluorescence image of an area with advanced mineralization in MC3T3-E1 culture with 1 mM βGP at 5.5 weeks after plating. The optical slices were acquired in the middle of a mineralized nodule parallel to the well surface. Sparse osteocyte-like cells (Oc) embedded into mineralized ECM (M, blue autofluorescence) have relatively short and less abundant processes (arrowheads) compared to osteocytes in parietal bone and CBL (cf. Fig. 4A,B). Their deficient morphology, scarcity and lack of extensive interconnected network are consistent with deficient upregulation and low expression levels of late osteoblast and osteocyte genes *Dmp1*, *Mepe*, and *Sost* in MC3T3-E1 cultures (Fig. 2 and Supp. Fig. S3). (*D*) Compositions of mineralized ECM nodules in MC3T3-E1 cultures vs compositions of CBLs in PBC cultures (βGP and Rap as indicated). The compositions were measured by Raman microspectroscopy at 11 weeks post confluency as described in Fig. 8.

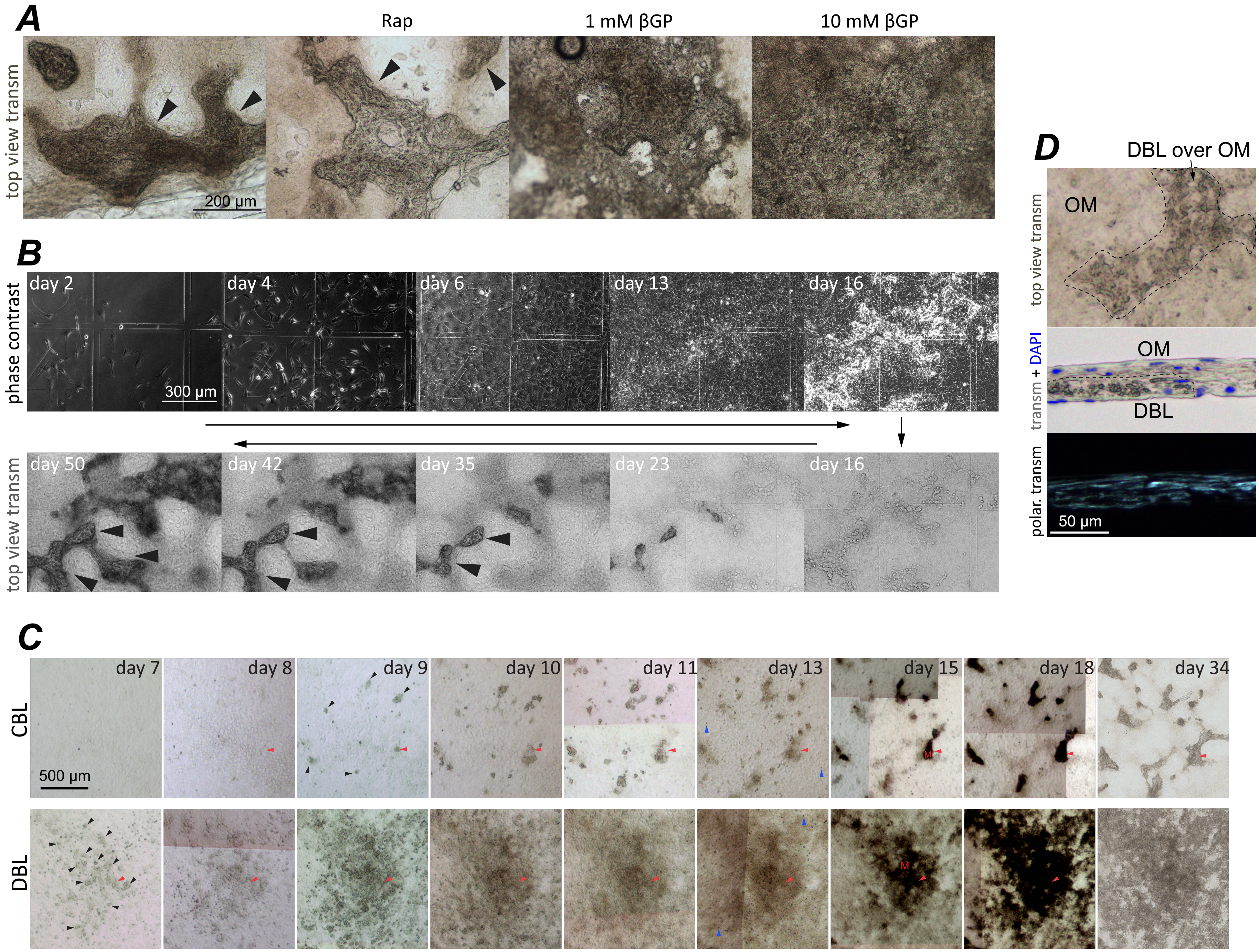
A
Rap
1 mM βGP
10 mM βGP
top view transm
200 μm
B
phase contrast
day 2
day 4
day 6
day 13
day 16
300 μm
top view transm
day 50
day 42
day 35
day 23
day 16
C
CBL
DBL
day 7
day 8
day 9
day 10
day 11
day 13
day 15
day 18
day 34
500 μm
M
D
DBL over OM
OM
top view transm
transm + DAPI
OM
DBL
polar. transm
50 μm

Supplemental Figure S5. Formation and growth of CBL and DBL cell-ECM structures in cultures of primary PBCs. (*A*) Effects of indicated supplements on top view appearance of CBL structures at advanced growth stage (10X/0.3 NA objective). Arrowheads point to lateral osteoid. Color balance and intensity of the bright-field transmission images were corrected as described in Methods.

(*B*) Growth of CBL structures monitored by imaging the same cell culture area with phase contrast (days 2-16 after plating) and transmission (days 16-50). The culture was expanded at reduced (5%) O_2 until reaching confluence and transferred to atmospheric (~ 20%) O_2 at day 6 as described in Methods. It was then treated with 10 nM Rap for a week (days 6-13). Formation of dense osteoblast clusters was observed at days 13-16. Mineralization became clearly visible at day 23 followed by growth of mineralized spicules and formation of trabecular-like structures through day 50. Note that location of many future CBL spicules can be identified based on osteoblast islands formed by day 16.

(*C*) Growth of CBL and DBL structures within the same culture well monitored by time-lapse transmission imaging at indicated days after plating (no-βGP/no-Rap). Black arrowheads mark initial clusters of plump cells (possibly dividing osteoblast precursors or early osteoblasts) which precede formation of mineralized nodules (cf. similar plump cells at day 16 in panel *B*). Much higher cluster density was observed in DBL than in CBL. Red arrowheads mark the same spots on the culture well at which plump cell cluster are observed. In CBL, plump cells appear at the spot only once (day 9), cover increasingly larger area (days 9-11), and then become invisible (day 13). Formation and progression of the cluster is accompanied by ECM deposition, which is followed by nucleation of mineralization (day 15) and lateral nodule growth (days 15-34). In DBL, plump cells appear at the same spot at least twice. The first appears at day 7 and becomes invisible at day 8. Another cluster appears at day 9 and becomes invisible at day 10. Thick ECM becomes apparent at day 13 and initial mineralization at day 15. High density, overlaying and overlapping of the bone-forming nodules arising from these clusters appear to cause disorganized formation and prevent well-defined lateral growth of DBL nodules (days 15-34). Blue arrowheads mark dying and dead cells. The images at days 7-18 were tiled from snapshots of overlapping culture well areas taken with a 4X/0.1NA objective, registered to each other using fiducial marks (well surface defects and entrapped dust particles), subjected to the same linear contrast enhancement, and cropped to desired areas. As a result, some of the cropped images were composed of multiple, tiled snapshots. The images at day 34 were taken with a 10X/0.3NA objective and corrected for color balance and intensity as described in Methods.

(*D*) Top view and cross-section images of an extended OM structure with mineralized islands overlaying OM within the area demarcated by dashed lines (day 34 after plating, no-βGP/no-Rap). Mineral within the islands was bone-like apatite based on Raman spectra (not shown) and appeared simultaneously (days 15-18) with mineralization of extended DBL areas in the same well (panel *C*). The adjacent OM remained unmineralized through day 34. Morphology of the islands, mineral composition, and the time course of their appearance suggest that they are DBLs overlaying OM.

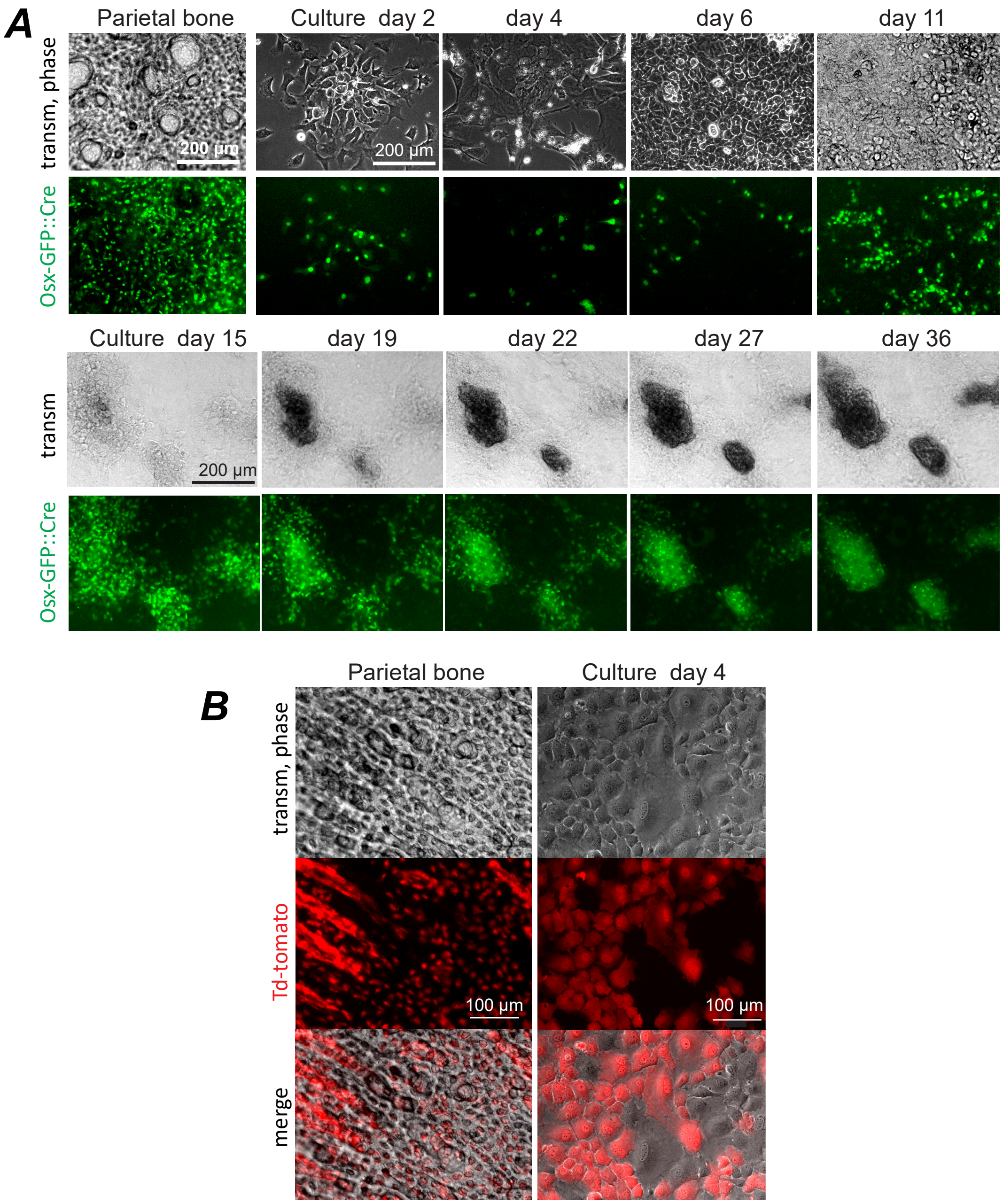

Supplemental Figure S6. Visualization of osteoblasts in cultures of primary PBCs during formation and mineralization of cell-ECM structures and in parietal bone from 3-6 day old mice. (*A*) CBL formation by primary PBCs from Osx-GFP::Cre mice, in which *Sp7* (osterix) expression is visualized based on nuclear localization of GFP::Cre fusion protein (green fluorescence) expressed under the control of *Sp7* promoter (no βGP or Rap). Multiple green cells were observed in clusters on day 2 after seeding. The fraction of green cells was reduced during proliferation at 5% O_2 (days 2-6). The culture was transferred into a standard incubator (~ 20% O_2, 5% CO_2) after reaching confluency on day 6. Subsequent cell proliferation and differentiation was accompanied by formation of thick clusters with increased number of green cells (days 11-15), which formed mineralized CBL spicules (days 15-36). Hazy green background is autofluorescence of mineralized ECM within these spicules. (*B*) Labeling of mature osteoblasts and their progeny by irreversible, conditional activation of tdTomato transgene (red fluorescence) in parietal bone and culture of primary PBCs (no βGP or Rap, 5% O_2) by Cre-recombinase expressed under the control of a *Bglap* (osteocalcin) promoter (see Methods).

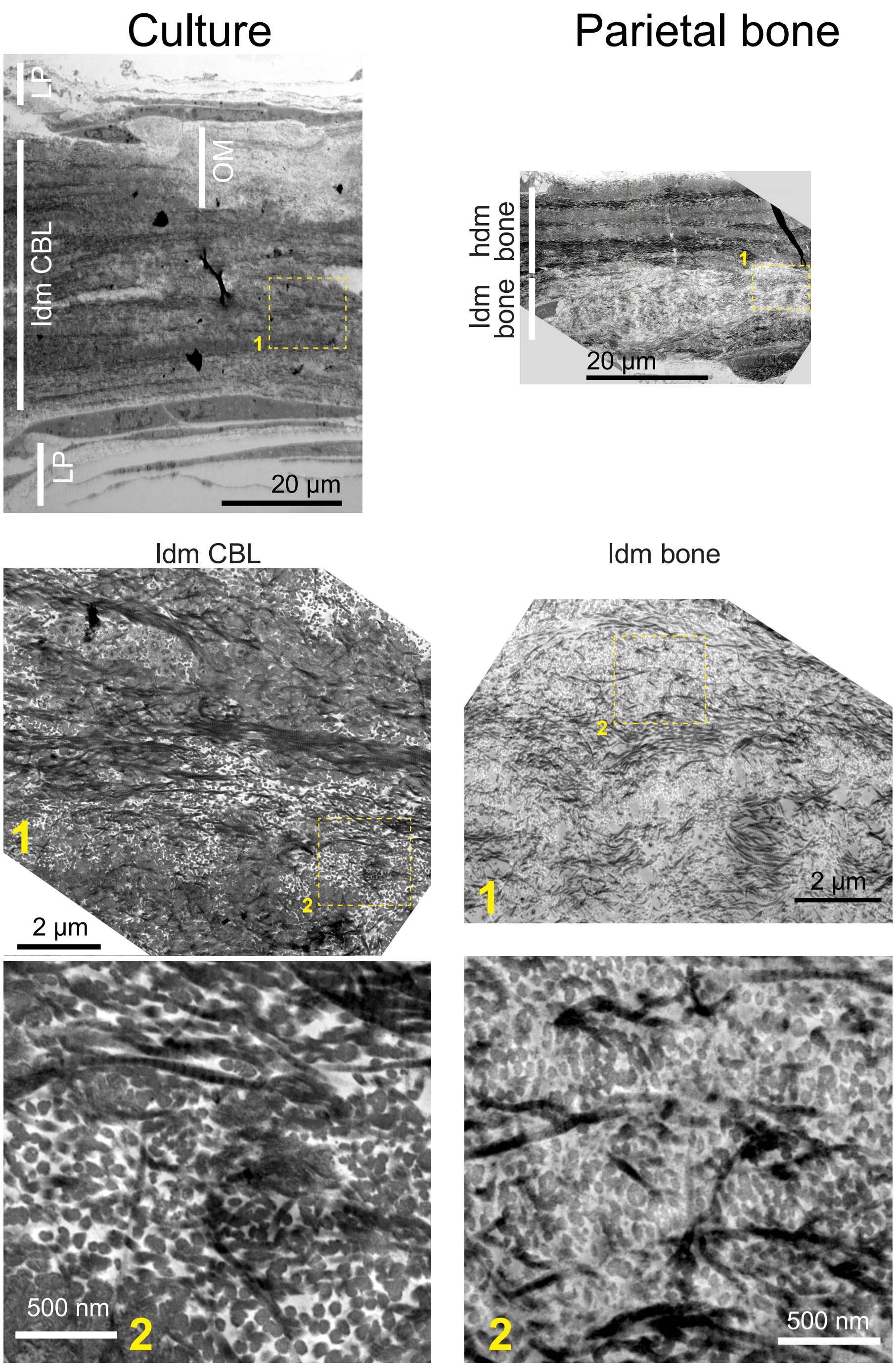

Supplemental Figure S7. TEM images of lower density matrix (*ldm*) within CBL in PBC culture (no βGP or Rap) and within parietal bone from a 4-day-old mouse (c.f. higher density matrix, *hdm*, in main text Fig. 4C). Low resolution images (top row) show layering of LP, *ldm* CBL, and OM structures in culture and layering of *ldm* and *hdm* in bone. Boxes mark successively zoomed-in regions shown in the correspondingly numbered panels.

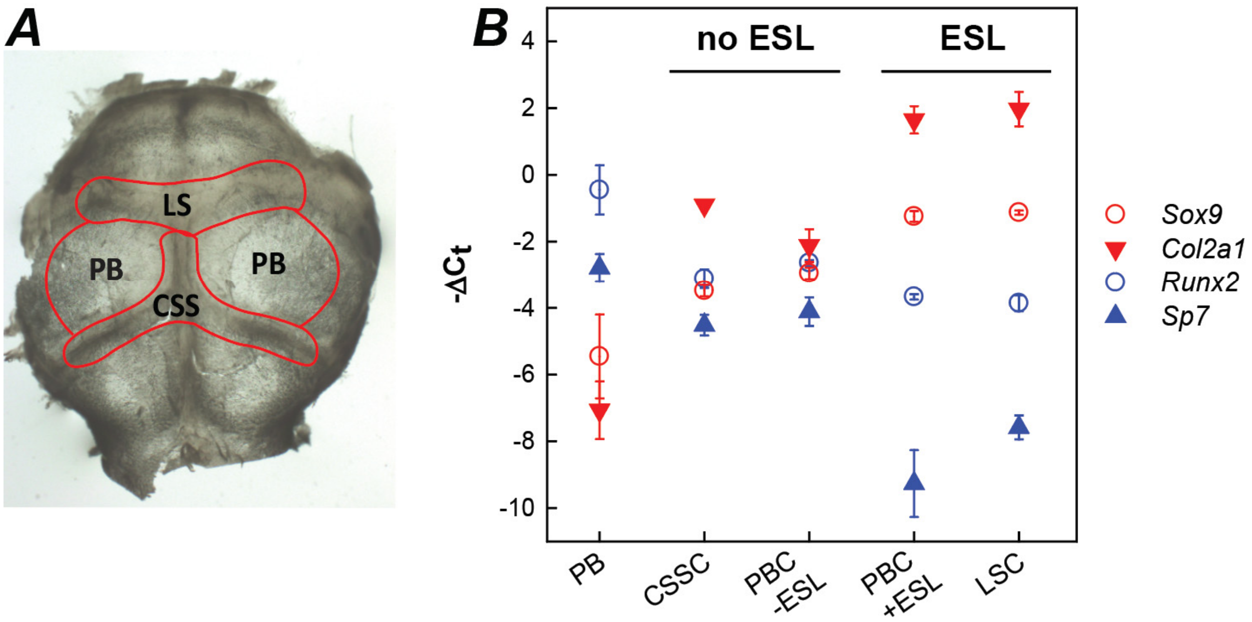

Supplemental Figure S8. Chondrogenic differentiation in ESL cell-ECM structures. (*A*) Parietal bone (PB), coronal and sagittal suture (CSS), and lambdoid suture (LS) areas used for establishing the corresponding PBC, CSSC, and LSC primary cell cultures from mouse calvaria. (*B*) Expression of chondrogenic (*Sox9*, *Col2a1*) and osteogenic (*Runx2*, *Sp7*) differentiation marker genes in parietal bone *in vivo* (PB, 17-day old mice, *N*=11 animals) and in primary cell cultures (*N*=3 wells) established from the calvaria regions shown in (A). PBC +ESL (PBC culture that formed ESL), CSSC, and LSC cultures were from the same preparation (N=6 animals pooled together). PBC -ESL (PBC culture that did not form ESL) was from an independent preparation (*N*=21 animals pooled together). Error bars are standard deviations.

Since independent preparations (isolations from animals) of primary PBC cultures produced highly variable amounts of ESL and some did not produce ESL at all, we suspected that ESL in primary PBC cultures could result from variable contamination with suture cells. We therefore established the four separate cultures of cells from parietal bones and their surrounding sutures described above. All cells were seeded at the same density (3,000 cells/cm^2) and maintained in the growth media supplemented with Asc2P (no-βGP/no-Rap) at 37 ^oC, 5% O_2 for 8 days (2 days past confluency). At this timepoint, RNA was harvested for qPCR from 3 wells for each type of culture. The other wells were transferred to atmospheric O_2 (~ 20%), to confirm the presence or lack of ESL.

We observed that LSC and PBC +ESL cultures formed ESL starting from ~7 days after the transfer, whereas CSSC and PBC -ESL did not form ESL by day 30 after the transfer. By the latter timepoint, all the cultures formed the other types of cell-ECM structures (LP, OM, CBL and DBL), which sometimes overlayed ESL. The CSSC culture (which lacked ESL) formed predominantly OM and LP with small areas of CBL/DBL. ESL was more extensive in LSC than in PBC +ESL culture.

Compared to *in vivo* PB, all cultures had reduced *Runx2* and *Sp7* and increased *Sox9* and *Col2a1* expression. Yet, only PBC +ESL and LSC cultures containing ESL had dramatic downregulation of *Sp7* (~ 50-fold) and upregulation of *Col2a1* (~ 500-fold), indicating chondrogenic differentiation. Taken together, these observations suggest either PBC and LSC culture contamination with chondrocyte progenitors (e.g., from periosteal/endosteal membranes continuously covering parietal bone and adjacent occipital bone cartilage) or inconsistent chondrogenic differentiation of progenitor cells (which could be enhanced by the incubation at 5% O_2).
